# Chromosome Folding Promotes Intrachromosomal Aberrations under Radiation- and Nuclease-Induced DNA Breakage

**DOI:** 10.3390/ijms222212186

**Published:** 2021-11-10

**Authors:** Yuri Eidelman, Ilya Salnikov, Svetlana Slanina, Sergey Andreev

**Affiliations:** 1 Mathematical Biophysics Laboratory, Emanuel Institute of Biochemical Physics of RAS, Kosygin Str. 4, 119334 Moscow, Russia; ushwood@yandex.ru (Y.E.); tetrahc@yandex.ru (I.S.); svetlana.slanina@gmail.com (S.S.); 2 Department of Semiconductor Quantum Electronics and Biophotonics, PhysBio, National Research Nuclear University MEPhI, Kashirskoye Shosse 31, 115409 Moscow, Russia

**Keywords:** chromosomal aberrations, Hi-C, HTGTS, polymer modeling, 3D chromosome organization, contact-first mechanism, structure heterogeneity, breakage-first mechanism, ionizing radiation, recurrent DSBs

## Abstract

The long-standing question in radiation and cancer biology is how principles of chromosome organization impact the formation of chromosomal aberrations (CAs). To address this issue, we developed a physical modeling approach and analyzed high-throughput genomic data from chromosome conformation capture (Hi-C) and translocation sequencing (HTGTS) methods. Combining modeling of chromosome structure and of chromosomal aberrations induced by ionizing radiation (IR) and nuclease we made predictions which quantitatively correlated with key experimental findings in mouse chromosomes: chromosome contact maps, high frequency of cis-translocation breakpoints far outside of the site of nuclease-induced DNA double-strand breaks (DSBs), the distinct shape of breakpoint distribution in chromosomes with different 3D organizations. These correlations support the heteropolymer globule principle of chromosome organization in G1-arrested pro-B mouse cells. The joint analysis of Hi-C, HTGTS and physical modeling data offers mechanistic insight into how chromosome structure heterogeneity, globular folding and lesion dynamics drive IR-recurrent CAs. The results provide the biophysical and computational basis for the analysis of chromosome aberration landscape under IR and nuclease-induced DSBs.

## 1. Introduction

The influence of spatial genome organization on chromosomal aberrations (CAs) is a problem attracting attention for many years. The genome is packed into discrete chromosomal territories. How the 3-dimensional organization of highly variable territories [1,2,3] determines CAs and selects the pathways of cancer recurrent translocations remains a hot topic of research in molecular and cancer biology [4,5,6,7]. Two main mechanisms of CAs are discussed, contact-first and breakage-first [5,8,9,10,11,12]. The key difference concerns which of the following matters, pre-existing contacts (contact-first) or delayed contacts formed in the course of the movement and interaction of damaged loci (breakage-first). In a contact-first mechanism, the formation of translocations is limited to neighboring chromosomes, broken chromosome ends cannot freely diffuse and translocations are formed from closely spaced DSBs. This may be due to factors that hold broken chromosome ends in place. The experiments with knockdown of the DNA-end-binding protein Ku80 caused a loss of the positional stability of broken chromosome ends and allowed them to move in nuclear space [13]. In this way, the breakage-first mechanism may be activated. Regardless of whichever mechanism prevails, the spatial proximity of loci in the genome should increase the probability of inter- and intrachromosomal aberrations under the influence of various DNA-damaging factors, including ionizing radiation (IR) [7,14,15].

Intrachromosomal proximity is a factor which influences the formation of translocations [12,16,17]. CAs measured by the multicolor banding (mBAND) [18,19] reveal the non-random position of loci which underwent exchange, or breakpoints, along the chromosome. These data suggest the proximity effect in the chromosome conformation or non-random spatial organization of chromosomes. New perspectives in the mechanistic study of CAs arose from the development of high-resolution genome-wide methods of exploring the chromosome structure and CAs, chromosome conformation capture (Hi-C) and high throughput genome translocation sequencing (HTGTS) [7,20,21]. The combined Hi-C and HTGTS approach was applied to IR and nuclease-induced translocations [5,21] in the mouse genome. The correlation between the 3D organization of chromosomes and the distribution of translocation breakpoints was uncovered [5,21].

The understanding of inter-relation between chromosome structure and aberrations is complicated by the unexplored role of chromosome structural variability and heterogeneity in the CA formation. Individual chromosomes maintain partly a domain organization on a megabase scale and exhibit variable structure on a large scale [1,2,3]. To track structural fluctuations of chromatin configuration in single cells different approaches were applied, including polymer modeling, single-cell Hi-C, FISH [1,2,3,22,23]. However, the origin of heterogeneity and single-cell variability of the chromosome architecture in a cell population remains poorly studied [22]. Therefore, the role of these factors in CA formation is still unclear.

In radiation cytogenetics, chromosome aberrations of translocation type are defined as a type of interchromosomal exchange aberrations between different non-homologous chromosomes. In the literature describing the methods detecting position of rearrangements, such as HTGTS, intra- and interchromosomal exchanges or translocations are often not separated terminologically [5,20,21]. To reconcile the terminology, we use in this work the term cis-translocations, or simply translocations for intrachromosomal exchange aberrations.

CA modeling enables the quantitative elucidation of underlying mechanisms. The simulation of 3D organization of chromosomes is one of the central points in mechanistic modeling of CAs. To generate chromosome structures, specific algorithms were developed. In approach [24,25], the nuclear volume was divided into cubic boxes and chromosome territories were modeled as a subsequent occupation of the closest neighboring boxes. The chromosome model in the form of a random walk (RW) or Gaussian chain, i.e., polymer without excluded volume interactions [26], was used to predict parameters of IR-induced CAs [27,28]. In the polymer approach [29,30,31], 3D structure of chromosomes was generated using the algorithm that takes into account the inter-chromosome interactions in the nucleus [32]. The simulated structures of chromosome territories were applied to calculate intra- and interchromosomal aberrations induced by IR with the different track structures [30]. For intrachromosomal aberrations induced by γ-irradiation, a more simple approach was introduced, where the ensemble of structures of individual chromosomes was obtained upon the suggestion that chromosome folding was due to interactions between subunits within the chromosome [33].

Aberration modeling approaches mentioned above share a common internal weakness, an inability to validate the models of 3D chromosome organization from current structural approaches, such as Hi-C. On the other hand, experimental information on translocations obtained by genome-wide HTGTS mapping together with Hi-C contacts analysis [21] was not yet understood in terms of biophysical mechanisms. To address these issues, we developed the polymer modeling approach allowing the reconstruction of 3D chromosome conformations based on genomic data and predict the characteristics of CAs in murine cells. This approach was applied to a detailed joint analysis of structural and aberration data from Hi-C and HTGTS methods. We demonstrated that the heteropolymer globule was a structural principle of chromosome organization in mouse cells which was the best consistent with Hi-C data. It was established how the single-cell variability and heterogeneity of the 3D organization of chromosomes in a cell population affected the breakpoint distribution in different chromosomes. We provided evidence that cis-translocation breakpoint distribution was determined by both the spatial proximity of loci at the moment of irradiation and the subsequent movement of damaged loci. The results provided new insight into biophysical mechanisms of cis-translocations under IR- and nuclease-induced DNA breakage and demonstrated the predictive power of the proposed computational approach.

## 2. Results

### 2.1. Modeling of Chromosome Aberrations Based on Polymer Physics of Chromosomes

**Figure 1:**
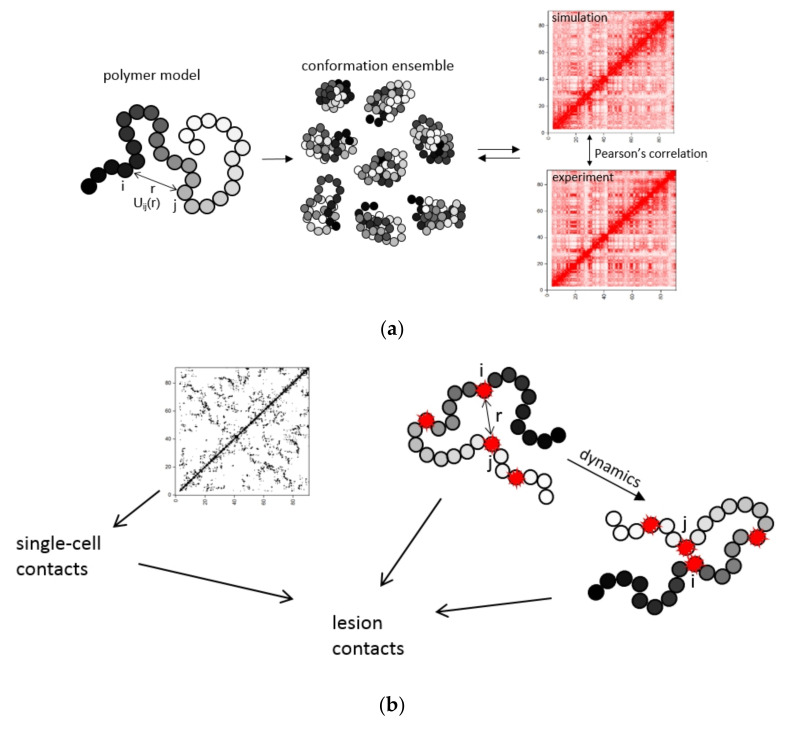

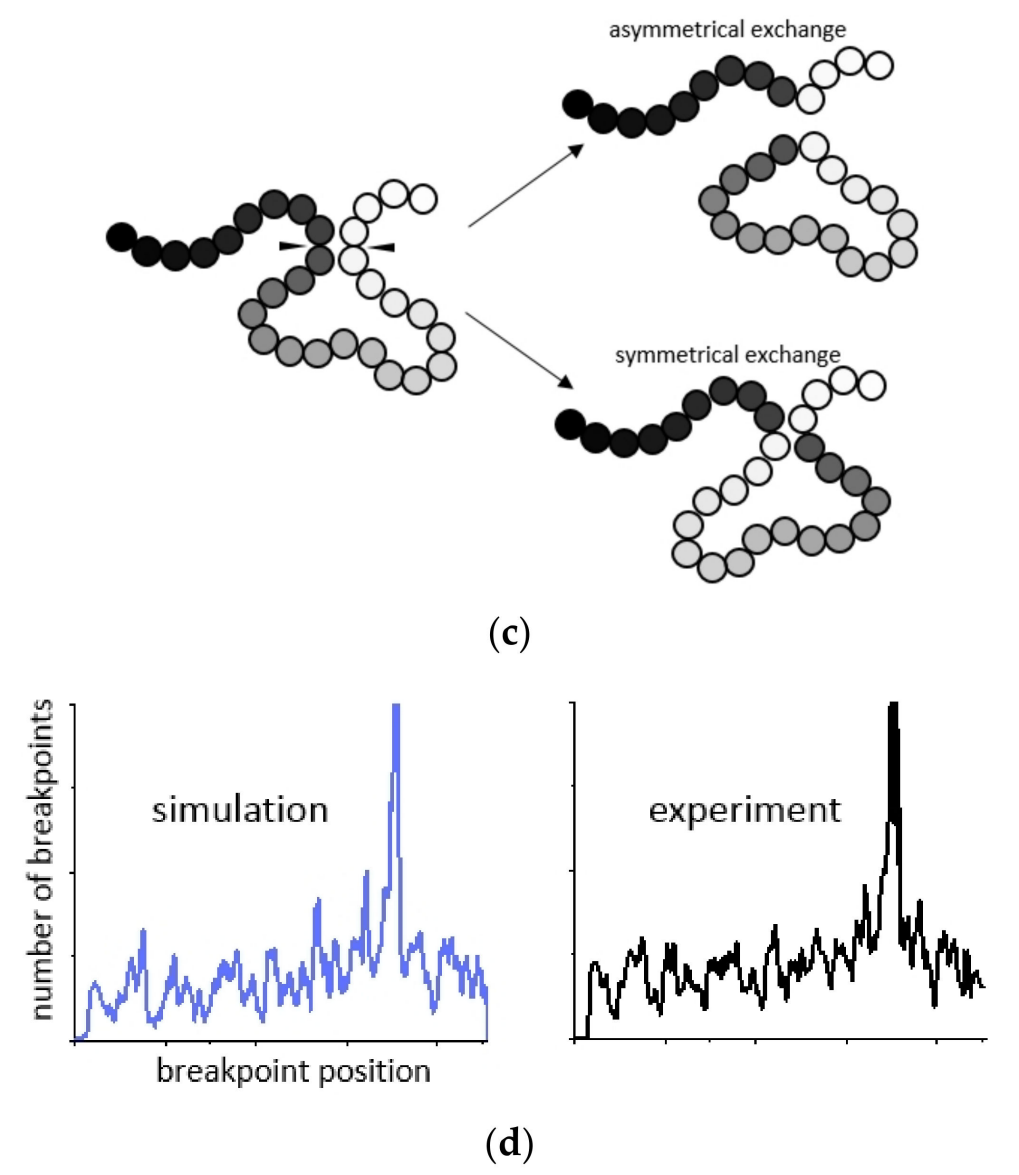
CA modeling based on physics of chromosome organization. (**a**) The structure of an interphase chromosome simulated by a heteropolymer globule model. A statistical ensemble of 3D chromosome conformations was generated and optimized by the iterative description of population-averaged experimental Hi-C contact frequencies. (**b**) Simulation of spontaneous, IR and nuclease-induced (recurrent) DNA double-strand breaks (DSBs) along the chromosomes. Damaged loci are indicated by red asterisks. The optimized ensemble was used to calculate single-cell contact maps and 3D distance distribution between damaged loci (lesions). Modeling chromosome lesion dynamics. (**c**) Simulation of intrachromosomal exchange aberrations, cis-translocations. The place of contact of the lesions is designated in arrowheads. An aberration occurs when two damaged loci are in contact at the moment of irradiation (contact-first mechanism) or come into contact after irradiation with time (breakage-first mechanism). (**d**) Calculation of the population-averaged distribution of aberration breakpoints both from single-cell contact maps and the spatial heterogeneity function, 3D distance distribution between lesions. Data analysis.

We developed an approach to CA modeling which extracted information about 3D chromosome structure organization from Hi-C data and applied it to the evaluation of CAs. This approach allowed the determination of both population-averaged and single-cell characteristics of intrachromosomal contacts and aberration breakpoint distributions in different chromosomes. Figure 1 shows the strategy of the proposed approach.

To probe chromosome aberrations, we built a physical model of a chromosome (Figure 1a). To this aim, we introduced the potentials of volume interactions between non-neighboring chain monomers (Figure 1a). The size of monomers was chosen according to the maximum resolution of experimental contact maps for the mouse genome, 100 kb [21]. We introduced the heteropolymer chromosome model with attraction potentials different for every pair of monomers [34]. It resembles the approach for chromatin submegabase domain simulation [23]. It was modified and applied to the scale of entire chromosomes. Following structure simulation with the use of molecular dynamics, we obtained a thermodynamic ensemble of structures (see description in Methods). The optimization of potentials went on until the iteratively calculated and average contact frequency measured by Hi-C became highly correlated (Figure 1a).

To quantify CAs and obtain the distribution of initial DNA damage or subunit lesions along the chromosome (Figure 1b) three types of DNA double-strand breaks (DSBs) were incorporated into the model, IR-induced, nuclease-induced, and spontaneous. Since IR-induced and spontaneous DSBs are stochastic in nature, their distribution along chromosomes was modeled by the Monte Carlo technique. For γ-radiation, the number of IR-induced DSBs in chromosome at a given dose D was sampled from the Poisson distribution. To reconstruct spontaneous DSB distribution in chromosome, which is not available in the experiment [21], unlike the spontaneous aberration breakpoint distribution, we developed the iterative method based on the comparison between the spontaneous breakpoint frequencies predicted from the tentative DSB distribution and the experimental frequencies (see Methods).

CA characterization requires knowledge of 3D position of damaged chromosomal subunits (Figure 1c). Experimental genome sequencing approaches, high-throughput translocation sequencing HTGTS [20] and chromosome conformation capture Hi-C were developed to map contacts and aberration positions on chromosomes [21]. However, these methods are population-averaged with limited information on single-cell variability of contact frequency and heterogeneity of spatial distances between chromosome regions in individual cells [22]. Physical modeling provides the missing data and maps spatial positions and contacts of nondamaged and damaged loci in individual chromosomes within a cell population. This creates an opportunity for a more comprehensive analysis of CAs than available on the basis of the HTGTS technique.

Hi-C and HTGTS methods offer a static view on interactions and don’t capture the dynamics of chromosome loci which presumably plays a role in CA formation. To explore the impact of locus movement on aberration formation we simulated the dynamics of damaged loci and estimated the contribution of delayed contacts, or the breakage-first mechanism, to the formation of cis-translocations (Figure 1c). In addition, the hypothetical scenario was studied that irradiation leads to a chromosome conformation change that affects contacts pattern, loci position and CAs frequency. Finally, the variety of chromosome contact and aberration breakpoint distributions obtained by both physical modeling and Hi-C/HTGTS were systematically analyzed.

The origin of an initial event responsible for an exchange aberration is a question of debate. Our hypothesis is that both loci containing DSBs are not converted into chromosome breaks but represent some kind of unstable lesion participating in exchange [8,35]. This hypothesis is supported by the finding that there is no significant change in the location of single DSB ends and broken ends are positionally stable [36]. An alternative approach [31] was based on the assumption that DSBs in a chromosome produce free chromosomal ends. At present, knowledge of the mechanism of chromosome rearrangement at the site of an exchange aberration is obscure. It is possible that both of these cases occur and result in different types of aberrations [11].

### 2.2. Physical Modeling of Hi-C Data Reveals Heteropolymer Globule Organization of Mouse Chromosomes

An important point in structure modeling is the assumptions about monomer types and interaction potentials. Previous models postulated two [37] or six [38,39] types of interacting monomers in chromosomes to explain the observation of a checkerboard pattern of chromosomal Hi-C contact maps [37,38]. In [40], a constrained-based modeling algorithm was applied, where the distance between subunits is strictly related to the frequency of contacts. However, this approach is too simplified and not appropriate from the polymer physics’ point of view. Hi-C focuses on small distances between loci, and its patterns can be very different from those for the mean or median distances. And two loci being further apart on average can have higher Hi-C contact frequency [41].

Here we examine the new chromosome model which introduces heterogeneous interaction potentials between any non-neighboring monomers, heteropolymer globule model, thus avoiding ad hoc postulates (block-copolymer structure, uniformity, types, etc.) which may result in biased predictions. The heteropolymer globule model takes heterogeneity of interactions into account accurately. Physical modeling of the chromosome provides a statistical ensemble of 3D structures and predicts the full spectrum of chromosome conformations in a cell population.

**Figure 2:**
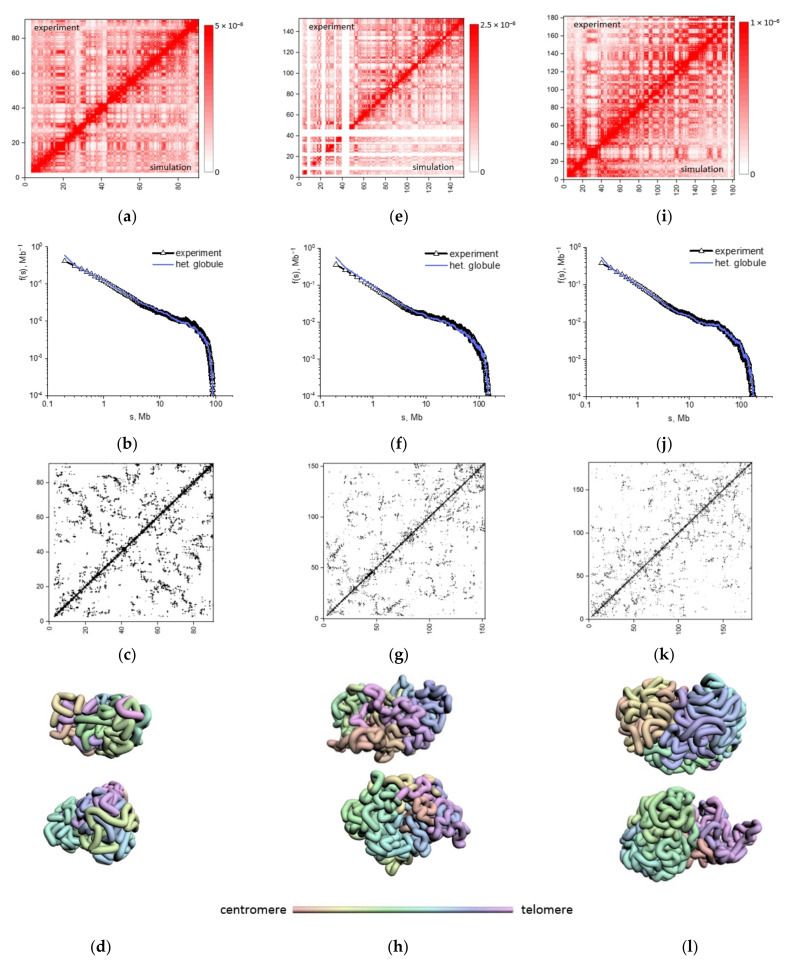
Globular organization of mouse chromosomes in G1-arrested pro-B cells. (**a**–**d**) Chromosome 18. (**e**–**h**) Chromosome 7. (**i**–**l**) Chromosome 2. (**a**,**e**,**i**) Hi-C maps, resolution 100 kb. Upper triangle: experiment [21]; lower triangle: simulation for the heteropolymer globule model of (**a**) chromosome 18, Pearson’s correlation R = 0.968; (**e**) chromosome 7, R = 0.951; (**i**) chromosome 2, R = 0.958. (**b**,**f**,**j**) Contact frequency as a function of genomic separation s, experiment compared to simulations by the heteropolymer globule model. (**c**,**g**,**k**) The single-cell contact maps for simulated chromosomes 18, 7, 2. Three individual cells are shown, the heteropolymer globular chromosome model. (**d**,**h**,**l**): Sample chromosome conformations in the optimized ensemble of structures. Chromosomes 18, 7, and 2. Coloring indicates the locus position along the chromosome.

Based on the heteropolymer globule model, we obtained the optimized thermodynamic ensemble of conformations for mouse chromosomes studied, 2, 7, and 18. The model predictions vs. the experimental measurements of contact frequencies argued that the heteropolymer globule chromosome model recapitulated experimental chromosome conformation capture data with high Pearson’s correlation (R = 0.951–0.968) (Figure 2a,e,i). The dependence of contact frequency on genomic separations f(s) for the heteropolymer model in the entire interval of genomic separations is highly correlated with the experimental dependence for all chromosomes (Figure 2b,f,j), Pearson’s correlation of f(s) for heteropolymer model and experiment is: for chromosome 18, R = 0.988; for chromosome 7, R = 0.987; for chromosome 2, R = 0.984. Contact frequency function does not follow a simple dependency f(s)~s^ν^ with the power coefficient independent of the interval of s.

High-quality description of the population-averaged chromosome interaction heatmaps served as a rigorous ground for solid prediction of the single-cell contact maps (Figure 2c,g,k) unavailable in Hi-C/HTGTS assay [21]. The single-cell contact maps simulated for the heteropolymer globular model for chromosomes 18, 7, 2 are notably different from the population-averaged contact maps (Figure 2a,e,i). They show statistical fluctuations of intrachromosomal contacts in individual cells. Several samples of chromosome conformations from the optimized ensemble of structures are presented (Figure 2d,h,l).

The structural organization of chromosomes encrypted in Hi-C maps is not observed directly. To analyze the relationship between structure and contact patterns, we calculated structural characteristics for alternative models of chromosomes 18, 7, 2: a self-avoiding coil, an RW model without excluded volume effects, and a homopolymer globule (Figure S1). The results show a significant difference between the prediction of the alternative models (Figure S2) and the experimental contact data. The heteropolymer chromosome model demonstrates the best correlation with the set of structural Hi-C data.

Specifically, for G1-arrested pro-B mouse cells, the previous Hi-C analysis suggested that chromatin was folded in a fractal globule state [21]. That followed from the power coefficient ν (exponent), determined genome-wide, being equal −1.05 in the range of 0.5–5 Mb. To assess the argumentation in favor of fractality we reproduced the method of Hi-C data analysis [21] for mouse chromosomes 2, 7 and 18 separately and plotted contact frequency for each pair of loci vs. genomic separation for each chromosome (Figure S3). The power coefficient ν in the range of 0.5–5 Mb varied from 0.87 to 0.92 for different chromosomes (Figure S3a,c,e). Alternatively, for the contact frequency f(s) the power coefficient varied from 0.87 to 0.93 in the same range of genomic separations (Figure S3b,d,f). The values for the two methods were close to each other and differed from −1. These numerical examples show two things. First, the analysis for mouse cells [21], similar to the method for human cells [37], was based on merging the data from all chromosomes throughout the genome. Therefore, the conclusion about the power coefficient −1, or about a fractal globule, does not refer to any specific chromosome, but applies to some abstract averaged chromosome. Second, the power coefficient can argue about the polymer state of the chromosome only indirectly. The description of the Hi-C contact maps is a more important, if not the only, argument for the validity of the polymer model of chromosome structure. Up to date, there is no evidence of the quantitative description of whole chromosome contact maps by a fractal globule model.

The heteropolymer globule model demonstrates a high correlation of contact frequencies predicted and measured by Hi-C for mouse chromosomes. It would be desirable in the future to study the mechanisms of compartmentalization of contact patterns as well as to extend structure validation of the model by bringing in available FISH information.

The analysis of the previous models of chromosomes shows that the heteropolymer globular model is the most compatible with the Hi-C data. This model accounts for heterogeneity of potentials in the most general way. It does not include any assumptions about the blocks of elements, and therefore provides the most accurate description of heterogeneous ensemble of chromosome structures. Chromosome structure modeling forms the basis for the prediction of chromosomal aberrations and evaluation of underlying mechanisms.

### 2.3. Structural Organization of Mouse Chromosomes Promotes IR-Recurrent Aberrations

The effects of IR on the distribution of recurrent cis-translocations in repair-deficient G1-arrested mouse pro-B cells were extensively studied by Hi-C and HTGTS methods [21]. A dramatic increase in the frequency of breakpoints of IR-induced exchange aberrations, compared to recurrent-spontaneous aberrations, was discovered not only in the vicinity of the site of I-SceI recurrently induced DSBs (hereafter will be referred to as “the breaksite”) but along the entire length of the chromosome. The interpretation of this finding was that most of the entire chromosome containing an individual site of recurrent DSBs became a “hot spot” for cis-translocations between the IR-induced damage and the site of recurrent DSBs [5,21].

To assess what one can expect from the biophysical consideration, we calculated the distribution of translocation breakpoints along a chromosome according to the contact-first mechanism.

**Figure 3:**
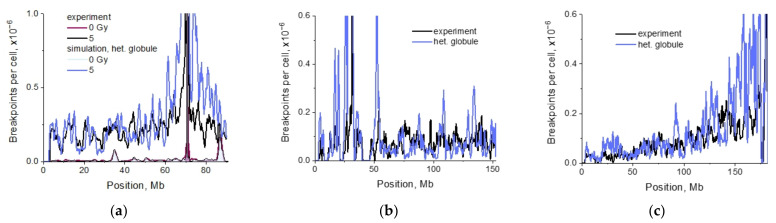
Cis-translocation breakpoint distributions in G1-arrested pro-B mouse cells evaluated by the contact-first mechanism. In all panels: experiment [21], simulations for the heteropolymer globule models. (**a**) Chromosome 18, the probability of contact-exchange P_c-e_ = 0.0029. For radiation doses of 0 and 5 Gy, Pearson’s correlation between theory and experiment R = 0.898 and R = 0.724, respectively. Following irradiation, the formation of translocations was taken into account both between recurrent and spontaneous, and between recurrent and IR-damaged chromosome subunits. (**b**) Chromosome 7, D = 5 Gy, P_c-e_ = 0.0029. (**c**) Chromosome 2, D = 5 Gy, P_c-e_ = 0.0035. For chromosomes 2 and 7, the control in [21] is negligible compared to 5 Gy, so it is not shown in the graphs. See Figure S4 for comparison with predictions for alternative models of structure.

The simulation of distribution of the cis-translocation breakpoints in mouse chromosome 18, obtained for the globular structures, predicted the effect of the high frequency of aberration breakpoints far from the breaksite. For the heteropolymer globule, correlation with experimental data [21] was R = 0.724 (Figure 3a). The correlations for homopolymer models (Figure S4b–d) are: for the homopolymer globule, R = 0.740, and for the loose globule, R = 0.720. The self-avoiding polymer coil poorly correlated with data, R = 0.586. In the absence of irradiation (control) the breakpoint distribution in chromosome 18 was consistent with the chromosome heteropolymer globule model (Figure 3a), R = 0.898. The calculations for the heteropolymer globule and the small dose 0.1 Gy as well as for the coil and large dose 5 Gy (Figure S5) indicated that the frequent breakpoints formed far from the breaksite disappeared in both cases.

For chromosome 7 (Figure 3b and Figure S4e–g) both the hetero- and the homopolymer globule models predicted high frequent breakpoints far from the breaksite, the polymer coil did not. Near the peak around the breaksite, the shape of the curve was described by the homopolymer globule and, surprisingly, by the model of the polymer coil.

Polymer modeling of chromosome 2 (Figure 3c) based on a heteropolymer globule model demonstrated no plateau and a gradual decline of the breakpoint frequency far from the breaksite. This prediction correlates with the data in the wide range of locus positions, from 0 to about 150 Mb. In the 30 Mb neighborhood of the breaksite, there is a deviation in the shape of the curves predicted and measured by HTGTS. The reason for that will be explored in the course of study of other CA mechanisms.

The modeling data demonstrated that the effect of high frequency of breakpoints along the entire chromosome was observed under certain conditions—hetero- and homopolymer globular state, frequent IR-induced damage (chromosomes 7, 18). The effect depended on a pattern of Hi-C contacts or chromosome compartmentalization non-trivially, and it was not observed for the heteropolymer globule model in chromosome 2. One can suggest that mechanisms other than contact-first may contribute to the shape of breakpoint distribution. Consideration of lesion dynamics together with conformational transitions would indicate, whether they predict the correlation of breakpoint distributions obtained by biophysical modeling and HTGTS.

Comparing the structural and aberration data on three studied chromosomes allows the determination of how the frequency of aberrations correlates with the level of chromosome compactness. The experimentally measured numbers of breakpoints per cell for chromosomes 2, 7 and 18 are 8.48 × 10^−5^, 5.38 × 10^−5^ and 9.78 × 10^−5^, respectively, which gives the ratio of 0.87:0.55:1. This demonstrates the same tendency as the ratio of the number of contacts in the heteropolymer globular model of chromosomes (Table S1) and in the Hi-C experiment (Table S2). Thus, the experimental data reveal that in the most compact chromosome 18, more intrachromosomal exchanges are formed than in less compact chromosomes 2 and 7. The simulations for the heteropolymer globule model show the same trend: numbers of breakpoints per cell for chromosomes 2, 7 and 18 are 1.45 × 10^−4^, 1.06 × 10^−4^ and 1.48 × 10^−4^, respectively, and the ratio is 0.98:0.72:1.

The results demonstrate that the contact-first mechanism of CA formation predicts the dependence of distribution of breakpoints on the 3D chromosome organization. It remains appropriate for qualitative description of the shape of breakpoint distribution in all chromosomes, including chromosome 2. CA model based on the heteropolymer globule principle is the only one which reconciles HTGTS and Hi-C data for all studied chromosomes.

### 2.4. Impact of Targeted vs. Nontargeted DSBs on Translocation Breakpoint Distribution

Here we use terms “targeted” and “nontargeted” DBSs. Targeted DSBs are recurrent DSBs induced at a fixed site by an endonuclease; nontargeted DSBs are induced randomly in the chromosome by γ-radiation. There are also spontaneous DSBs, which exist in certain non-random sites. They are targeted but not recurrent. One of the problems in the elucidation of the mechanisms of recurrent translocations is which factors determine the frequency of translocations and the distribution of breakpoints in the genome [4,5]. For recurrent translocations, it was concluded that the main contribution was made by targeted damage, a high frequency of recurrent DSBs at certain target sites in the genome [4]. For aberrations of the IR-recurrent DSB type, the observations suggested that the key role was played by the spatial proximity of loci in the chromosome [21]. This conclusion was based on the data on a single frequency of nontargeted DSBs, 5 Gy of γ-radiation. The important unresolved question is what is the contribution of targeted and nontargeted DSBs to the distribution of breakpoints, and how it depends on the structure of the chromosomes and IR dose.

**Figure 4:**
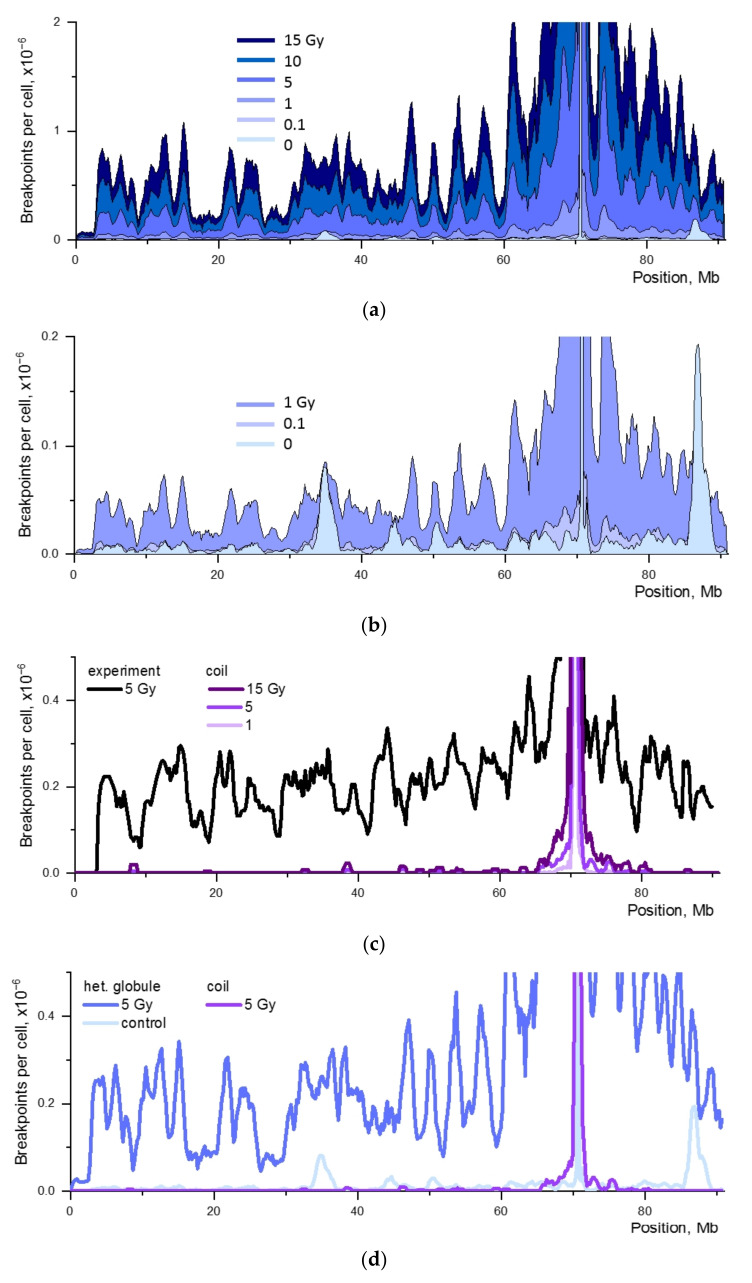

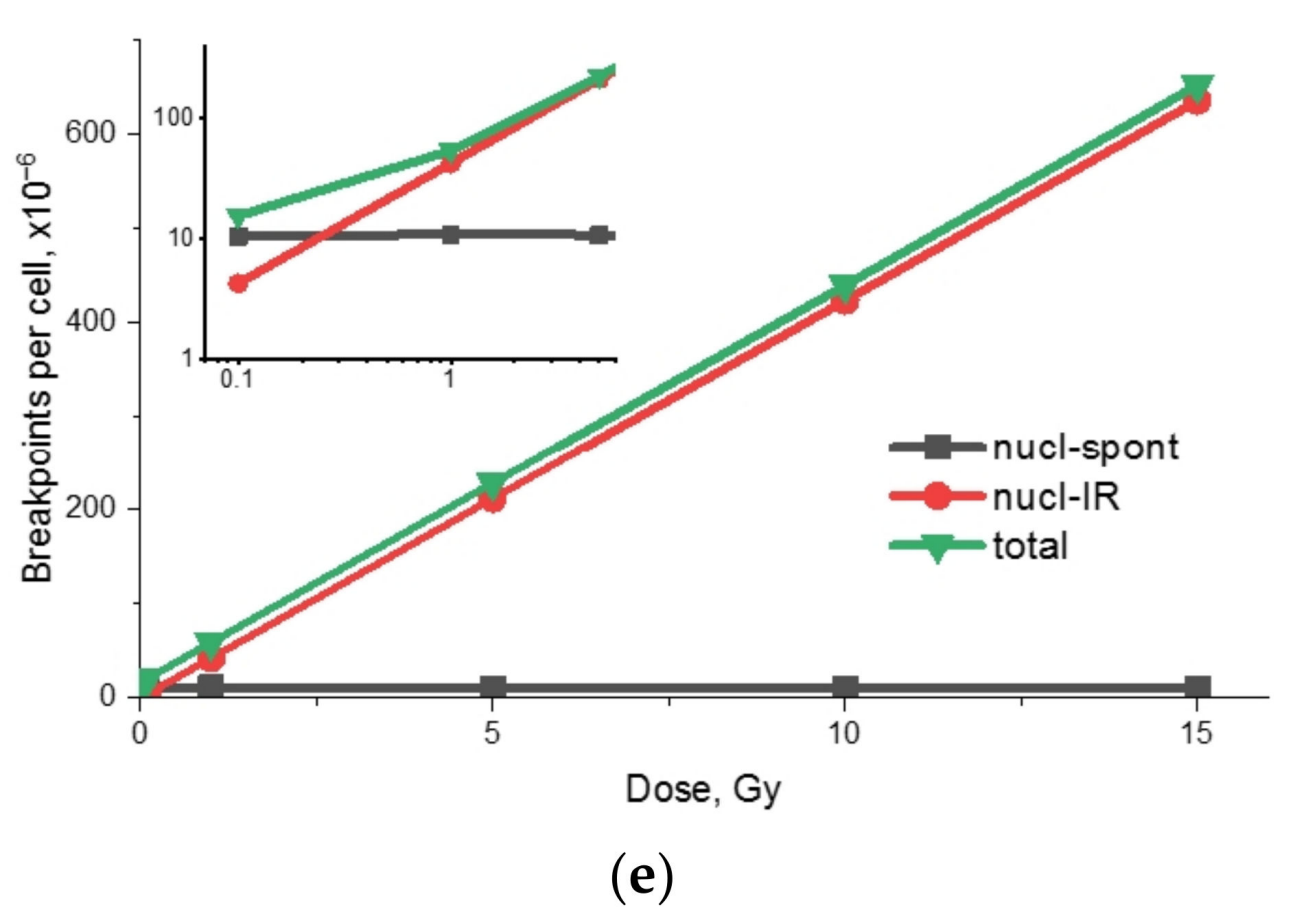
The impact of targeted and nontargeted DSBs on the formation of cis-translocation breakpoints. The calculations were made for chromosome 18 and the contact-first mechanism. P_c-e_ = 0.0029. (**a**,**b**) The chromosome model is a heteropolymer globule. Dose dependence of the breakpoint distribution. (**a**) Doses of 0–15 Gy. (**b**) Low doses (0–1 Gy) shown on an enlarged scale. (**c**) The breakpoint distribution for a polymer coil model, doses of 1–15 Gy. The experimental translocation breakpoint distribution for chromosome 18, D = 5 Gy [21]. (**d**) Breakpoint distribution for a heteropolymer globule and a polymer coil models of chromosome 18. D = 5 Gy and D = 0 (simulation for a heteropolymer globule model). (**e**) Dose response for the frequency of breakpoints of different origin. Abbreviations: nucl-spont, aberrations formed by the nuclease-induced and the spontaneous DSB; nucl-IR, aberrations formed by the nuclease-induced and the IR-induced DSB. The inset shows the low-dose range.

To find out the impact of targeted vs. nontargeted DSB frequency on translocation breakpoints, we calculated the breakpoint distribution in mouse chromosome 18 for different chromosome folding models, a heteropolymer globule and a decondensed polymer coil, in a wide range of frequencies of DSBs induced by radiation, 0.074–11.2 per chromosome corresponding to radiation doses of 0.1–15 Gy. The non-irradiated control, i.e., spontaneous and nuclease-induced DSBs, was taken into account. The calculated data comprise interaction of DSBs of different origins, both recurrent-IR and recurrent-spontaneous, under the contact-first mechanism of CA formation. The distributions of breakpoints for different doses are shown in Figure 4.

The relative contribution of targeted and nontargeted DSBs to the distribution of aberration breakpoints varied both with the radiation dose and with the large-scale organization of the chromosome. The simulations showed that the alteration in the shape of the breakpoint distribution with a change in the frequency of nontargeted DSBs (Figure 4) was not observed at doses higher than 5 Gy, in contrast to low doses. For doses of 0 and 0.1 Gy, the shape of the curve is determined by the interaction of recurrent and spontaneous DSBs. At the dose of 1 Gy, in distinct locus positions j purely spontaneous translocations remain significant (Figure 4b), but for most loci, the contribution of spontaneous aberrations to the shape of the breakpoint curve is low. The main factor determining the shape of the breakpoint curve at high doses, 5–15 Gy, is the spatial proximity of loci in the chromosome as a manifestation of its structural organization (Figure 4a).

We estimated the change in the shape of the breakpoint distribution at large genomic separations from I-SceI site depending on dose, provided that the structure of chromosome 18 was a coil rather than a globule. A very narrow peak in the immediate vicinity of the I-SceI site was observed in the distribution of IR-recurrent translocations, and the entire distribution was out of agreement with the experimental one (Figure 4c).

The results suggest that at high doses the shape of the breakpoint curve for chromosome 18 is described by the contact-first mechanism under the condition that the structure of the chromosome is a dense heteropolymer globule. At small doses (0.1–1 Gy), interactions of recurrent and spontaneous DSBs with each other, as well as with IR-induced DSBs, make a significant contribution to the shape of the curve. If the chromosome is a polymer coil, then the contact-first mechanism does not play a role in the distribution of breakpoints (Figure 4d).

The dose dependence of the total breakpoint frequency in chromosome 18 is close to linear (Figure 4e). At the same time, the radiation component is strictly linear, and the spontaneous component distorts the linearity at doses of 0.1–1 Gy. The dose linearity is due to the fact that we consider IR-nuclease exchange aberrations due to contacts of damaged subunits where one is formed by the nuclease-induced DSB regardless of the radiation dose and another is due to the IR-induced DSB. The dose dependence for frequency of IR-IR induced cis-translocations is determined by the frequency of paired contacts of damaged loci. It is described by the quadratic dose dependence (Figure S6).

Thus, the impact of mechanistic factors on the breakpoint distribution is determined by the balance of damage parameters, radiation dose (i.e., the frequency of induction of nontargeted DSBs), the parameters of the chromosome structure and, in the general case, the distribution of recurrent and spontaneous DSBs along the chromosome. This balance is accurately predicted by the proposed aberration model. At relatively low doses of γ-radiation the contact-first mechanism alone is not sufficient and spontaneous lesions should be considered.

### 2.5. Single-Cell Variability of Cis-Translocation Breakpoints

The aberration measurement by high-throughput translocation sequencing method HTGTS [5,20,21] provides a distribution of breakpoints along the chromosome. This information is population-averaged, obtained on a population of many millions of cells. It is not indicative of the single-cell variability of pairwise interactions and lesion contacts. How the breakpoints are distributed along the chromosome of a single cell remains obscure.

To study the pattern of translocations in individual cells we determined single-cell contact maps, distribution of contacts with the breaksite and of cis-translocation breakpoints for pools of different numbers of cells.

**Figure 5:**
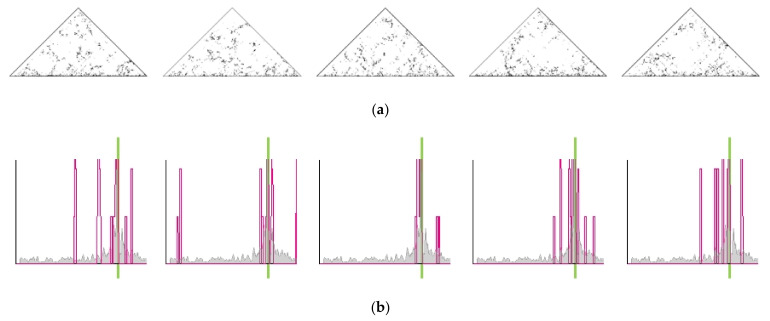

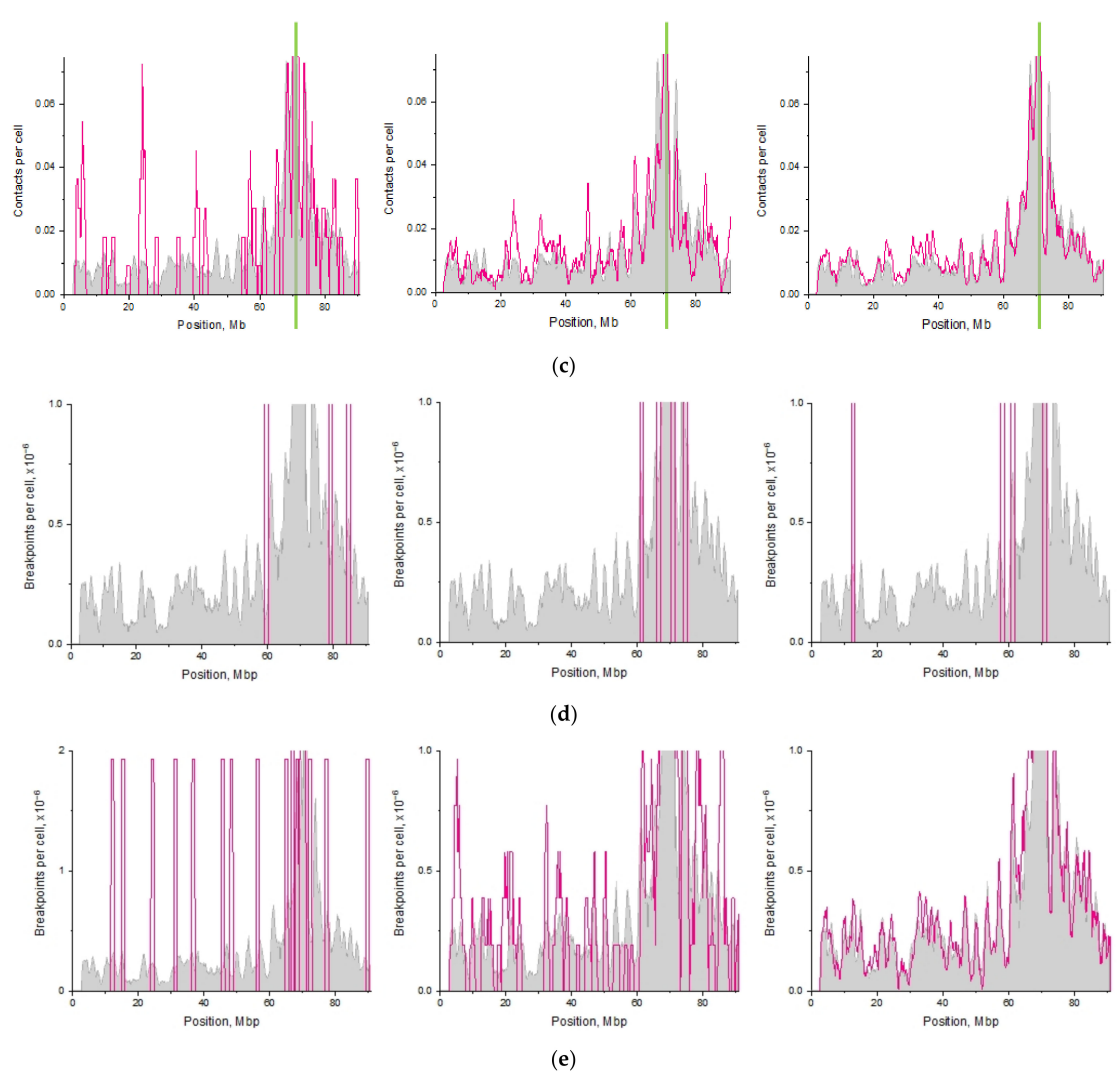
Single-cell, pooled and population-averaged distribution of contacts and translocation breakpoints. Chromosome 18, heteropolymer globule. (**a**) Single-cell chromosomal contact map, five individual cells. The black dots corresponding to the contacting pairs of loci are doubled in size for better visibility. (**b**) Single-cell distributions of the number of the contacts of any locus along the chromosome with the I-SceI site, five individual cells. The single-cell distribution is the sum of independent distributions for two chromosomes. (**c**) The distribution of the number of contacts (per cell) of any locus along the chromosome with the I-SceI site, averaged over a pool of 10, 100 and 1000 cells (left to right). The gray graph in panels (**b**,**c**) is the distribution of the number of contacts (per cell) of any locus along the chromosome with the I-SceI site, averaged over a pool of 2000 cells. The vertical green line denotes the position of the breaksite. The curves are smoothed as in the experimental study [21] (see Section 4). (**d**,**e**) Distributions along the chromosome of the number of breakpoints (per cell) averaged over a pool of a different number of cells. IR dose is 5 Gy. (**d**) 10 thousand cells, 3 independent repeats. (**e**) 100 thousand, 1 million, 25 million cells (left to right). The gray graph is the distribution for conventionally infinite cell population, averaged over a pool of 10^8^ cells (see Section 4).

The single-cell contact maps for the three chromosomes studied are presented (Figure 2g,k and Figure 5a). The fluctuations of intrachromosomal contacts are observed in individual cells. The single-cell distributions of the number of contacts of any locus along the chromosome with the I-SceI site for five individual cells indicate variability of position and frequency of contacts with the site of nuclease-induced DSBs (Figure 5b). Unlike single-cell distribution, the pooled distributions of contacts with the I-SceI site were averaged over the pools of 10, 100 and 1000 cells (Figure 5c). The gray graph in panels (b, c) is the distribution of contacts averaged over a pool of 2000 cells. The comparison shows how fluctuations of contacts are eliminated when the number of cells in the pool increased. The distributions of the number of aberration breakpoints per cell along the chromosome averaged over a pool of different numbers of cells at dose 5 Gy are presented in Figure 5d,e. In gray, the distribution for conventionally infinite cell population (averaging over 10^8^ cells) is shown. Strong variability of position and shape of breakpoints distribution is observed for pools of 10^4^–10^6^ cells with respect to the shape of the population-averaged curve (gray). With the increase in the number of cells in the pool, the distribution approaches the population-averaged one (the rightmost curve). For 25 million cells, the distribution is only weakly distinct from conventionally infinite cell population.

The distribution for chromosomal contacts with the breaksite is very different in scale from that for breakpoints (Figure 5c–e). The population-averaged distribution of breakpoints was obtained for 10^8^ cells, while distribution for contacts of chromosomal loci with the breaksite was for 2000 cells. This difference is explained by the values of two probabilities. The probability of IR-induced damage of a chromosomal 100 kb subunit and the probability of contact-to-exchange are small, 0.0041 at 5 Gy and 0.0029 respectively. The frequency of aberration breakpoints for a pair of contacting loci is roughly proportional to their product and is about 10^−5^. Formation of an aberration breakpoint is a significantly rarer event than formation of a chromosomal contact. For this reason, fluctuations in breakpoints increase dramatically, and to reach the population-averaged shape (gray), a much higher number of cells is required.

**Figure 6:**
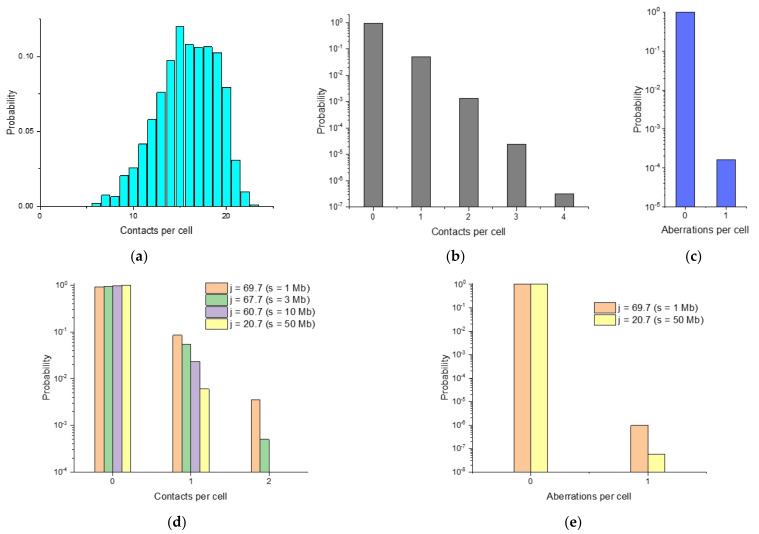
Probabilistic characteristics of contacts and chromosomal aberrations. Chromosome 18, heteropolymer globule. (**a**) Distribution of the number of contacts between chromosomal loci and the breaksite. (**b**) Distribution of the number of contacts between damaged loci and the breaksite. (**c**) Distribution of the number of aberrations, total for all pairs of loci. (**d**) Distribution of the number of contacts between the individual locus j and the breaksite. Locus positions and genomic separations from the breaksite are shown in the graph. (**e**) Distributions of translocation breakpoints for individual locus j. All distributions were calculated per cell, taking into account two copies of the chromosome in the cell. The distributions for contacts of damaged loci with the breaksite were calculated for D = 5 Gy.

Stochastic aspects of the formation of breakpoint distribution are also manifested in the fluctuation of the number of contacts, breakpoints, and aberrations at a given average value. Single-cell fluctuations may be characterized by the probability of a different number of contacts of damaged loci with the breaksite. Figure 6a–d shows the calculated distribution of the number of contacts of all loci with the breaksite, of all damaged loci with the breaksite, the distribution of the number of translocations per cell and the distribution of the number of contacts with the breaksite for the individual position of four loci. The distribution of the number of contacts of any locus with the breaksite shows the presence of multiple contacts (paired, triple, etc. up to 23 in one cell or in one pair of chromosomes) (Figure 6a), in the globular chromosome 18. Figure 6e shows the probability of the number of translocations for two of the loci presented in Figure 6d.

To quantify the impact of various factors (chromosome conformations, radiation damage, lesion interactions) on the fluctuation of breakpoints, we used the ensemble of chromosomal conformations and characterized the contribution of each factor by calculating relative fluctuations, the ratio of the standard deviation to the mean value, SD/mean. For the model of chromosome 18 in the form of a heteropolymer globule and a radiation dose of 5 Gy, this was done for the following values: the total number of contacts of intact loci in the globular chromosome, the number of contacts with the breaksite, the number of contacts of damaged loci with the breaksite, the number of breakpoints. The results are summarized in Table 1.

The results demonstrate that in individual cells, unlike the “population-averaged” one, the fluctuations of any characteristics, including contacts and damage, take place. The drastic increase in relative fluctuations is observed for the number of breakpoints with respect to fluctuations in the number of contacts in the chromosome. Fluctuations depend on the parameters of the structure, chromosome subunit damage and aberration formation. According to the calculations, the greatest relative fluctuations are achieved for the number of breakpoints (~110).

Thus, the main factors responsible for the strong variability of translocation breakpoints in single cells are the fluctuation of the number of damaged subunit contacts with the breaksite and the low probability of contact-to-exchange event.

### 2.6. Heterogeneity in Spatial Chromosome Organization among Individual Cells and Breakpoint Distributions

Studying the role of heterogeneity of spatial chromosome organization in the distribution of breakpoints is complicated by the methodological issue. Pseudo-4C distributions used to correlate chromosome structure and aberration breakpoints measured by HTGTS [5,21] do not contain information on heterogeneity of 3D distances between damaged loci in individual chromosomes. This information might be obtained by imaging approaches (like FISH), but such data are not available for the studied cell line and for any loci required. However, even if they were available, it would remain unclear how heterogeneity of spatial distances in a cell population is related to the heterogeneity of breakpoint distribution along the entire chromosome.

**Figure 7:**
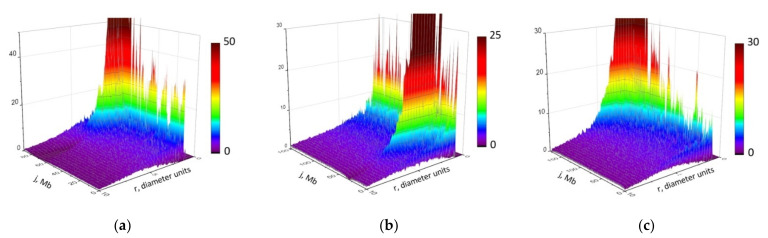
Quantification of IR-recurrent damage heterogeneity in different mouse chromosomes. Damage heterogeneity function φ_i,j_(r;D) for γ-radiation. D = 5 Gy. Locus i is the position of the I-SceI recognition site, r is the distance between locus i and damaged locus j with DSB of any origin, spontaneous or IR-induced. Three-dimensional graphs of φ_i,j_(r;D) for chromosomes 18, 7, 2, the heteropolymer globule model. (**a**) Chromosome 18, (**b**) chromosome 7, (**c**) chromosome 2. All panels show the distance distribution of chromatin lesion pairs (i,j) per cell, 2φ_i,j_(r;D).

To overcome this problem, we found a quantitative relationship between the translocation breakpoint distribution and spatial heterogeneity of chromosome organization in the ensemble. To this aim, we introduced functions describing the heterogeneity of all (ψ_i,j_(r)) and damaged (φ_i,j_(r;D)) chromosome locus pair distances in the cell population. The definition and the physical sense of these functions are detailed in Methods, Section 4.1. The distance distributions ψ_i,j_(r) for individual loci are shown in Figure S6 as calculated for different chromosome models. The complex shapes of damage heterogeneity function φ_i,j_ for chromosomes 18, 7, 2 are shown in Figure 7 for γ-radiation dose 5 Gy. Figure S8 demonstrates the distance dependence of φ_i,j_ function for individual loci with different genomic separations from the breaksite. The results present the analysis of heterogeneity distribution φ_i,j_ for different polymer models of chromosomes.

Figure 7 shows the 3D landscape of function φ_i,j_(r;D) calculated with the heteropolymer globule model for different mouse chromosomes at D = 5 Gy. φ_i,j_(r;D) at doses 0.1 and 15 Gy for chromosome 18 is demonstrated in Figure S9. At a relatively low dose of 0.1 Gy, there are differences in the shape of the distribution curve of the distances between the damaged loci from the calculations for 15 Gy. This is explained by the increased contribution of spontaneous damage at 0.1 Gy.

To quantify the breakpoint frequency by means of structure spatial heterogeneity we defined the number of breakpoints per cell between loci i and j damaged at dose D, f_bp_(i,j;D) through the integral on distribution of distances between the damaged loci (see Section 4, Equations (12) and (13)). The physical sense is that spatial heterogeneity of damaged subunits, usually being considered at arbitrary distances, is manifested in breakpoints at distances of damage colocalization necessary for CA formation. How the spatial heterogeneity of DNA damage shapes cis-translocation breakpoint frequency is demonstrated for different chromosomes (Figures S10 and S11a–c).

With respect to the single-cell contact approach (Figure 3), we get almost the same Pearson’s correlations between the experiment and simulations. The close association (Figure S11d) of two predictive approaches (single-cell contacts vs. distance heterogeneity function) was possible only if the Hi-C contact distance (R_cont_), or a distance of the maximum size of the region of contact detectability, was equal to the size of lesion colocalization area (R_cont,CA_) necessary for CA formation. Otherwise, they would provide different predictions for breakpoint frequency. This, in turn, would complicate HTGTS data analysis by the necessity of their systematic correction for heterogeneity. More study is required in the future to understand the difference in two approaches in greater depth.

Here we assumed that the optimized ensemble of structures obtained by physical modeling fully reflected the heterogeneity of chromosome 3D organization in a cell population. This is a simplification and one cannot exclude different independent subpopulations and unknown factors contributing to the spectrum of chromosome conformations. Here we explored a physical version of the origin of structural heterogeneity with a minimum of postulates.

The above predictions of CA breakpoint distributions by two approaches are limited by consideration of the pre-existing contacts, or the contact-first mechanism. The approach based on 3D distance distributions is extended to take the dynamics of damaged loci into account in the next paragraph.

### 2.7. Translocation Breakpoint Distribution Depends on Movement of Damaged Loci

Partial disagreement between breakpoint distributions predicted according to the contact-first mechanism and the data (Figure 3 and Figure S11) for individual regions or chromosomes can be explained by neglecting a number of factors. One of them is the movement of damaged loci after irradiation. Here we evaluated the contribution of damaged locus dynamics to CA frequency, i.e., the breakage-first mechanism. We assumed that mobility of damaged loci induced by γ-radiation did not differ significantly from the mobility of nondamaged loci. Locus dynamics in chromosome, assessed as the mean squared displacement as a function of time, was validated against the experimental data [42] on dynamics of IR-damaged loci for mammalian cells (Figure S12).

To model CAs with incorporating dynamics of damaged loci, we quantified the contribution of contacts of damaged loci at various times after irradiation. To consider pre-existing (t = 0) and dynamic contacts (t > 0), the modifications were introduced to the previous calculation algorithm. Instead of the probability of contact-exchange, the rate of conversion of contact into exchange was introduced (V_c-e_). We also introduced a new function describing the number of contacts between damaged loci at different initial distances from each other, formed in the interval t, t + dt. The integral of this function over a time interval (0, t) gives the number of contacts at different initial distances from each other, formed during this interval (see Section 4). The calculations were made with the following approximation. During the modeling of locus dynamics in chromosomes, for damaged loci, the first contacts formed were converted into aberrations with rate V_c-e_. The likelihood that the same lesion contact which avoided CA formation could decay and form again after some time was neglected. This approximation as well as observing lesion dynamics for the period of time from t = 0 to 3.3 h were due to large computational costs. We also assumed that for repair-deficient murine pro-B cells in which aberrations were measured [21], the DSB repair could be neglected. I. e. the number of damaged loci was not changed due to DSB repair during locus movement. And the cis-translocations were formed due to misrepair of contacted damaged loci.

**Figure 8:**
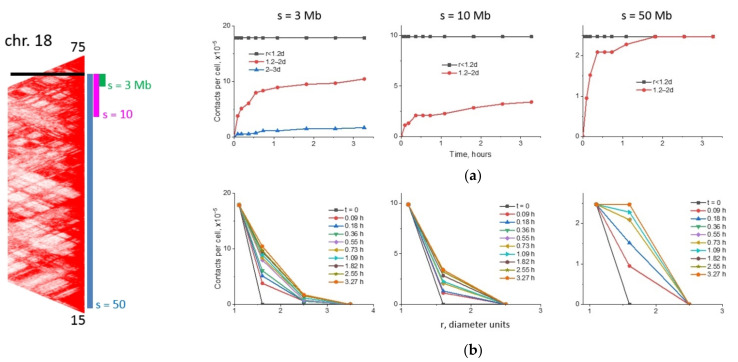

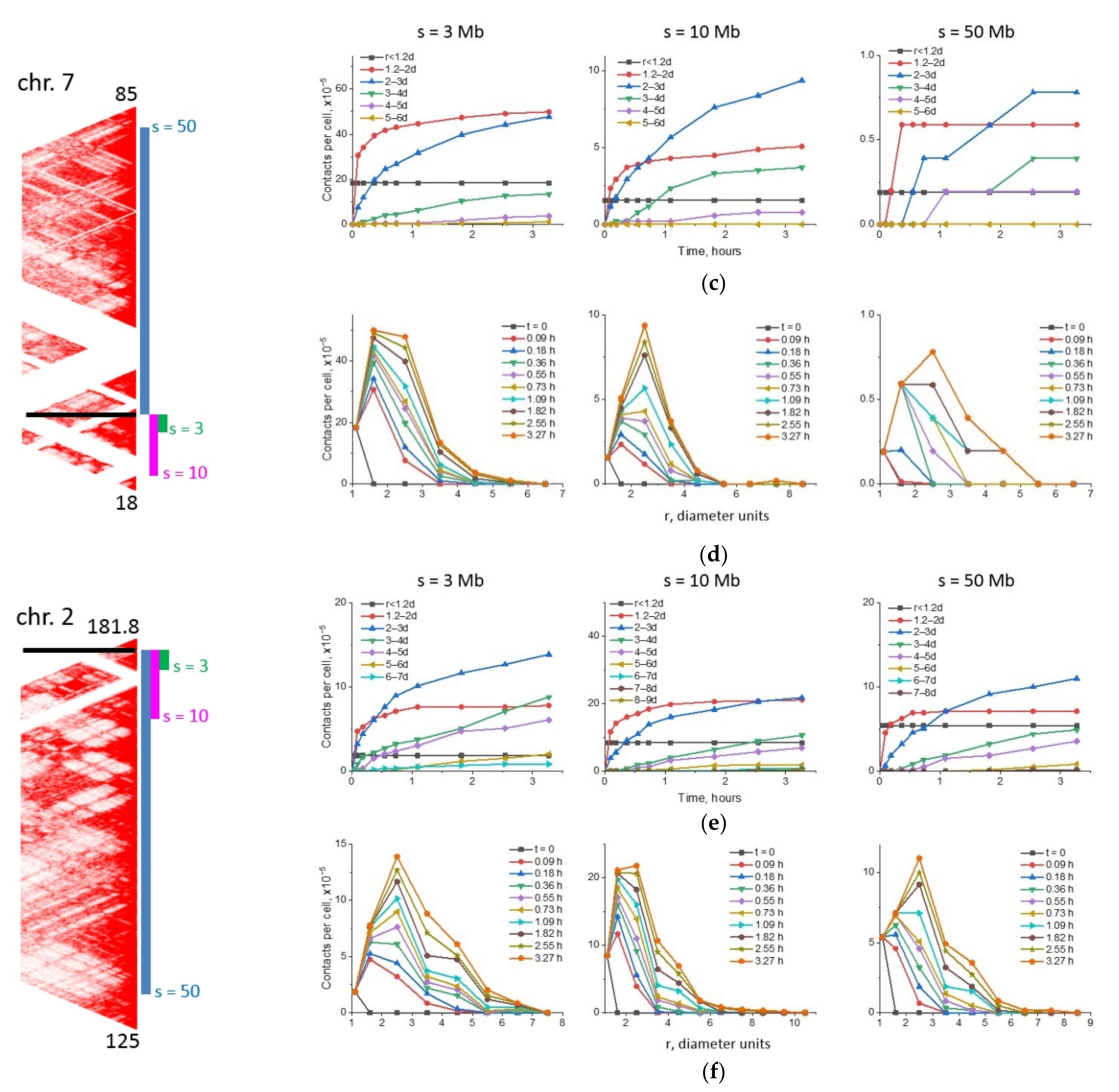
The simulated dynamics of lesion contact formation. Left side: The segments of the calculated contact maps including the loci under consideration. The heteropolymer globule model of chromosomes. The numbers above and below are the genomic positions of the segments, Mb. The black lines indicate the positions of I-SceI site. The colored stripes to the right of the map show the genomic separations between the considered loci. Graphs: the examples of dynamics of locus pairs with genomic separations s = 3, 10 and 50 Mb from I-SceI site and at the spatial distance r, r + dr at the initial time. γ-radiation dose D = 5 Gy. (**a**,**b**) Chromosome 18; (**c**,**d**) chromosome 7; (**e**,**f**) chromosome 2. (**a**,**c**,**e**) The number of contacts formed during the time from 0 to t between the damaged loci located at different initial distances between them, in diameter units. (**b**,**d**,**f**) The number of contacts formed as a function of the initial distance between the damaged loci for different observation times t (observation interval from 0 to t).

The simulated dynamics of lesion contacts, both differential, in the interval t, t + dt (Figure S13) and their integral, over time (0, t) (Figure 8) is presented for different chromosomes.

We observed for chromosome 18 (Figure 8 and Figure S13), which is the densest of the three (Figure S1, Table S1), that the number of additional lesion contacts formed over time is significantly less than the number of initial contacts at the moment of irradiation. This applies to all three genomic separations presented and to all initial distances between lesions; only at the largest genomic separation s = 50 Mb, the lesions that were initially relatively close to the breaksite, at the distance of 1.2–2 d, make a contribution comparable to pre-existing contacts (i.e., with lesions initially located at the distance of up to 1.2 d). At the initial distance of more than 2 diameters, contacts are almost not formed at all. Therefore, there are very few lines in Figure S13a and Figure 8a (compared to chromosomes 7 and 2), and in Figure S13b and Figure 8b, the curves fall to zero very quickly. In more decondensed chromosomes 2 and 7, the new contacts are formed with a noticeable frequency in all considered time intervals and initial distances (Figure S13c–f). This leads to the fact that at both large and small genomic separations, by the time t = 3.3 h, more contacts are formed between the elements with initial distances of 1.2–2 d and 2–3 d than they were at the initial time (Figure 8c,e). In chromosome 2, at s = 3 Mb (Figure 8e), even the initial distances of 3–6 d (i.e., 2.5–5 times greater than the contact distance) make a noticeably greater contribution to the total number of contacts than pre-existing contacts.

**Figure 9:**
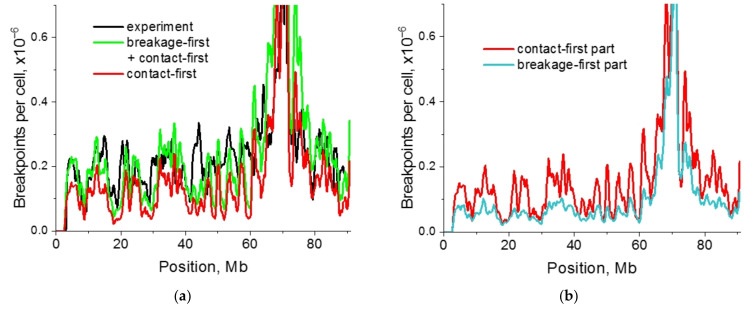

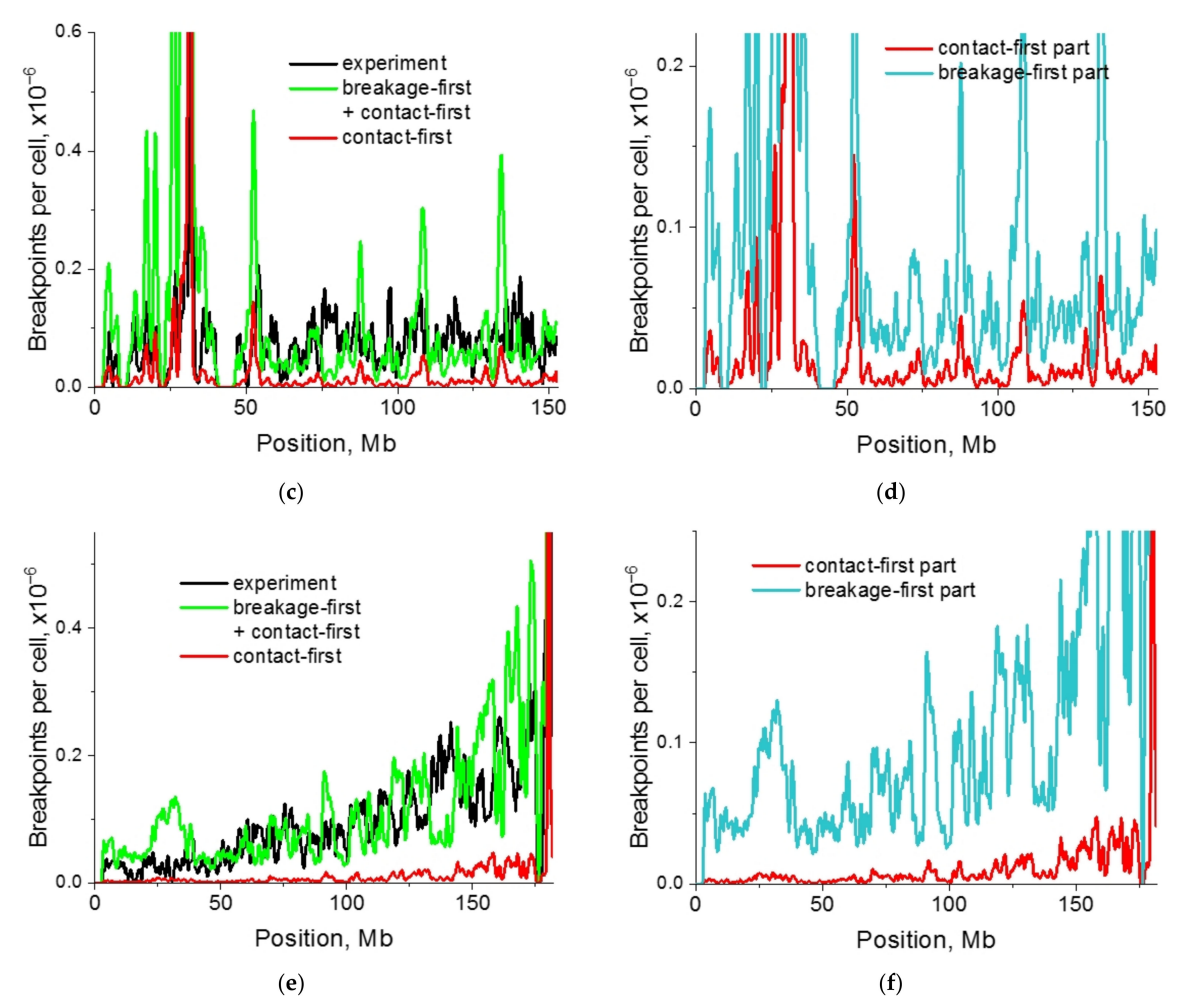
Distribution of breakpoints in mouse chromosomes 2, 7, 18 with contribution of pre-existing contacts and lesion dynamics. Dose of γ-irradiation 5 Gy. The heteropolymer globule model of chromosomes. (**a**,**b**) Chromosome 18; (**c**,**d**) chromosome 7; (**e**,**f**) chromosome 2. (**a**,**c**,**e**) Comparison of calculations on the breakage-first plus the contact-first mechanism (green curves) with the experiment. The contribution of the contact-first mechanism, i.e., translocations formed at t = (0, 0 + dt) is shown as red curves. (**a**) The rate of contact-exchange for chromosome 18 V_c-e_ = 1.54/h, (**c**) for chromosome 7, V_c-e_ = 0.52/h, (**e**) for chromosome 2, V_c-e_ = 0.28/h. Chi-square values for data description by different mechanisms are as follows. Chromosome 18: fitting only by the contact-first (Figure 3): Chi-square = 41.20; the breakage-first plus the contact-first: 29.25. Chromosome 7: the contact-first (Figure 3): Chi-square = 91.90, the breakage-first plus the contact-first: 76.55. Chromosome 2: the contact-first (Figure 3): Chi-square = 47.47, the breakage-first plus the contact-first: 41.04. (**b**,**d**,**f**) Impact of initial contacts (t = 0) and contacts formed at t > 0 on breakpoint distribution curves.

The numerical data (Figure 8 and Figure S13) characterize the heterogeneous dynamics of damage contacts in different chromosomes, illustrated by three loci with different genomic separations. The diffusion displacement of damaged loci depends on the local density of the subunits, which is determined by highly heterogeneous contact frequency maps (Figure 2). In chromosomes 2 and 7, the increased role of contact dynamics can be observed by the difference between the initial lesion contacts and the contacts at subsequent moments of time before the observation time (Figure 8). The dynamics of lesions may not end by 3.3 h. We cannot rule out that the role of lesion dynamics may be greater than estimated, at least, for less condensed chromosomes. Lesion contact dynamics coincide with aberration dynamics up to a constant. Thus, all conclusions considering the lesion contacts are also valid for the aberrations. The impact of the pre-existing contacts and lesion dynamics on the shape of the translocation breakpoint distribution in different chromosomes is demonstrated in Figure 9.

Accounting for the dynamics of chromosomal loci (Figure 9a,c,e) in CA formation resulted in the re-evaluation of the role of the contact-first mechanism. The number of breakpoints formed by the contact-first or the breakage-first mechanism (Figure 9b,d,f) differs for different chromosomes indicating their dependence on structural organization, i.e., on a contact map. For chromosome 18, our data suggest (Figure 9a,b) that the contact-first mechanism and dynamics of damaged loci contribute to the breakpoint distribution roughly equally. The extended plateau in breakpoint distribution for chromosomes 18 and 7 far from the breaksite is captured by both the contact-first and the breakage-first mechanisms for heteropolymer globule chromosome models (Figure 9a,c). Chromosomes 7 and 2 are looser than chromosome 18 (Figure S1, Table S1), and the role of lesion dynamics becomes more significant.

The modeling study of different mechanisms of CA formation (Figure 9 and Figure S14) implies that the correlation of chromosomal contacts and breakpoint frequency established by Hi-C and HTGTS [21] does not reflect the dominant role of pre-existing spatial proximity, or the contact-first mechanism, in the translocation formation. This statement is explained by the following observations.

The breakpoint distribution, predicted by the contact-first mechanism, is proportional to the distribution of pre-existing chromosomal contacts. In CA models without lesion dynamics (contact-first), aberrations formed at t = 0 are compared and fitted to aberrations at the time of observation. In CA models with lesion dynamics, aberrations are formed by the contact-first (t = 0) and the breakage-first mechanism (t > 0), then total aberrations (sum of both, at t = 0 and t > 0) are compared with and fitted to the aberrations observed. The contribution of the contact-first mechanism is determined quantitatively by subtraction of dynamically formed (t > 0) aberration breakpoint frequency from the total frequency (Figure 9 and Figure S14). The contribution to CAs from pre-existing chromosomal contacts (t = 0), therefore, depends on lesion dynamics (Figure 9 and Figure S14a–c, red curves). On the other hand, the shape of CA breakpoints for the contact-first mechanism is independent of lesion dynamics (Figure S14a–c, blue vs. red curves). Thus, the correlation between the breakpoint distribution predicted by the contact-first mechanism and the total distribution from two mechanisms reflects the similarity of the distribution shapes and not the quantitative contribution to the translocation frequency.

Incorporation of the movement of damaged loci resulted in reduced discrepancy between the predicted and the experimental breakpoint distributions, but did not eliminate it (Figure 9a,c,e). The difference between the mechanistic prediction and the experimental observation [21] remains for chromosome 2 in the 150–180 Mb range in the neighborhood of the breaksite and for chromosome 18 in about 20 Mb vicinity of the breaksite, for chromosome 7 local differences exist along the chromosome. One of the reasons may be the usage of the simplest suggestion that the rate of contact-exchange is independent of locus position. Experimental information about the rate of conversion of a damage contact to an exchange aberration as a function of locus position along the chromosome is missing. In this regard, the simplest assumption used may be reasonable to provide the first mechanistic conclusions.

Another possible explanation is the existence of an additional mechanism of locus displacement. This mechanism might comprise the conformational change of the structure following irradiation as a genome response to DNA damage [11,33]. We evaluated this hypothesis by computer simulations of chromatin conformational transitions. The numerical experiments demonstrate that the change of chromatin conformation at different scales decreases the disagreement between the theoretical predictions and HTGTS translocation profile significantly (Figure S15). The open question remains about the existence of conformational transitions following irradiation for repair-deficient mouse cell lines [11,33,43,44]. The alternative explanation is that the HTGTS method itself together with I-SceI endonuclease and IR triggered DSBs may induce changes in chromatin conformation in the extended neighborhood of the recognition site. It is in this area that there are differences between the observations and the predictions of CA model with both the contact-first and the breakage-first mechanisms (Figure 9).

In sum, we presented the first mechanistic prediction of IR-recurrent high-resolution translocation breakpoint distributions for G1-arrested mouse chromosomes. The theoretical distributions correlated with the observations made by two high-throughput genomic methods, Hi-C and HTGTS. Unexpectedly, theoretical vs. Hi-C/HTGTS correlations were achieved only under the certain principle of structural organization of chromosomes, the heteropolymer globule. A more deep analysis of translocation formation on the basis of mechanistic modeling is necessary although it is complicated by increasing uncertainty in the required information.

## 3. Discussion

To explore the impact of chromosome organization on the formation of cis-translocations we developed a physical modeling approach and presented for the first time the biophysical analysis of high-throughput genomic data from chromosome conformation capture (Hi-C) together with translocation sequencing (HTGTS) methods. The modeling strategy allowed to establish the relationship between chromosome structure, locus dynamics and cis-translocations induced by IR and a nuclease. The highly accurate description of Hi-C contact data for three mouse chromosomes revealed the new physical model of chromosome organization, a heteropolymer globule. The comparison of current and previous modeling analysis for mouse genome, both structural and aberrational, demonstrated that the chromosome model based on the heteropolymer globule principle of the organization was the best and allowed successful integration of information from high-resolution genomic methods, as well as provided prediction of chromosome aberration landscape under targeted and nontargeted DNA DSBs.

We determined the quantitative relationship between single-cell distributions for contacts and translocation breakpoints. Additionally, a method to infer translocation breakpoint distributions from the damage heterogeneity function, 3D distance distribution between damaged loci in the cell population, was introduced. These two different methods provided more deep insight into aberration breakpoint formation than available from experimental genomic approaches used in CA studies (Hi-C, HTGTS).

The results argued that the main factors responsible for the strong fluctuations of aberration breakpoints in single cells were the low number of lesion contacts and the low probability of a contact-to-exchange event. Our study uncovered how the globular chromosome folding and structural heterogeneity were manifested in the spatial distribution of pre-existing contacts and in lesion dynamics. These mechanistic factors drove IR-recurrent CA frequency and determined distinct breakpoint distributions in different chromosomes.

Finally, the possible implications and extensions of the proposed modeling approach should be outlined. DNA breaks in the genome are indispensable in major biological processes, such as cancer, aging, cell death. Our methodology focuses on the question of how the 3D organization of chromosomes contributes to genomic rearrangements in response to random (IR-induced) and recurrent targeted (nuclease-induced) DSBs.

The prediction of the dose-response for recurrent cis-translocation frequency showed a linear dependence for γ-radiation, consistent with the linear no-threshold concept. At low doses, there is a deviation from linearity. This is due to the contribution of spontaneous lesions and their interaction with the site of recurrent DSBs.

The implication of the model for the charged particle irradiation would be relevant in a wide area of radiobiological and biomedical research. Radiation with increasing linear energy transfer (LET) gives a more heterogeneous distribution of energy deposited in chromatin and chromosomes. This will lead to a heterogeneous spatial distribution of the damaged loci depending on the physical parameters of charged particle tracks and on the contact pattern in a chromosome. The model predicts the complex, multilesion contacts and complex aberrations for globular chromosomes. For γ-radiation, they are almost not observable, because damaged sites are relatively rare and dispersed. For particle tracks, the complex cis-translocations can be frequent. Many of them will be of a relatively small length and may escape from detection, being below the sensitivity threshold of experimental methods such as mBAND [12]. Our modeling approach can provide comprehensive information about intrachromosomal rearrangements induced by charged particle tracks, both detectable and hidden.

The aberration model reveals that the radiation-induced effect for γ-radiation at the chromosome level is influenced by the spatial organization of the chromosome, a contact pattern, locus dynamics. The calculations for the entire nucleus [45] revealed that the three-dimensional organization of the nucleus could affect the frequency of chromosomal aberrations caused by radiation due to the spatial distribution of contacts and the topology of chromosomes. For charged particles, the radiation damage effect at the chromatin level was dependent on 3D structure of the target [46]. A similar effect was observed experimentally for interchromosomal aberrations, the frequency of dicentrics was dependent on the architecture of the nucleus [47]. These findings suggest that the description of the radiation damage effect on the chromosomal/nuclear level is not a function of physical parameters only, such as LET or dose, but depends on 3D organization of chromosomes, interchromosomal interactions as well as on the microdistribution of energy deposited in nano-sized loci within each chromosome. This conclusion may touch upon the basic methodology of describing cellular radiation effects. Another interesting aspect that follows from the proposed aberration modeling approach is that still unrecognized characteristics of biological targets, their inherent single-cell variability and heterogeneity, may be of principal importance in the quantitative prediction of radiation effects for charged particles and γ-radiation.

The new methodology of 3D modeling of chromosome aberrations applied to a whole nucleus would account for interchromosomal interactions and may have application for prediction of aberration landscape in the course of IR-based therapy [45]. With the increase of particle stopping power, the frequency of lethal aberrations increases. However, the transformation ability of charged particles may also increase due to non-lethal aberrations, in particular, intrachromosomal rearrangements. This expectation is supported by a correlation of LET-dependence of relative biological efficiency (RBE) for cell neoplastic transformation and for intrachromosomal rearrangements [48]. If the correlation is causal, intrachromosomal aberrations can contribute to secondary cancers under irradiation of normal cells. On the other hand, intrachromosomal rearrangements after high-LET radiation can induce genome reprogramming in cancer cells and thus promote the appearance of a subpopulation of resistant cancer stem cells. The mechanisms of the response of tumor cells to genome damage by charged particles are poorly understood. In this regard, biophysical modeling may help to design well-defined explanatory experiments.

One of the possible extensions of our approach is to assessment of genomic rearrangements not only from IR-induced DSBs but from DNA breakage following chemotherapy treatment. Chemotherapeutic drugs induce S-dependent DSBs due to replication stress. In contrast to DSBs considered in the model and induced in G1 phase, replication-dependent DSBs may have different consequences on a spectrum of aberrations due to distinct mechanisms. A novel moment is that aberrations in a given cell cycle depend on the transfer of damaged chromosomes from the previous cell cycle and on the transformation of chromatid-type aberrations into chromosome-type in mitosis. The incorporation of replication-dependent DSBs and their transfer through the cell cycle in the modeling of chromosome aberrations has been initiated [49,50] and it relates to the problem of chromosomal instability. At the level of a three-dimensional chromosome organization, such a task has not yet been solved and remains a serious problem. In particular, it will require the prediction of Hi-C contacts in dividing cells. The next step would be the synthesis of both approaches, structure-oriented one (3D, Hi-C, etc.) and dynamical one [49,50] to account for mechanisms of IR-chemical damage induction, interaction and transmission through the cell cycle to the progeny of survived cells.

Among the perspectives of the present work is the modeling of translocations together with deletions, inversions, insertions, taking into account the 3D structure of the genome. The aim would be the usage of Hi-C data from multiple species for coherent analysis and mechanistic prediction of structural variations which are hallmarks of most cancer genomes. These types of rearrangements are now being extensively studied [51,52,53]. The distribution of somatic mutations is correlated with three-dimensional genome organization [53,54]. The developed mechanistic approach to 3D modeling of chromosome rearrangements can provide more deep information about mutation processes and their relationship to the 3D genome. These relationships may be important for identifying mutations in the genome of cancer patients.

In the presented modeling approach, we considered how chromosome folding determines cis-translocations under the action of targeted and nontargeted DSBs. The reversed question can be posed, how can the chromosome folding change under the DNA-damaging factors. This opens the way to modeling the genesis of cancer cells through the altered 3D genome folding as a response to replicative stress and other types of DSBs.

## 4. Materials and Methods

### 4.1. Polymer Modeling of Chromosome Structure

The structure of interphase mouse chromosomes 2, 7 and 18 was modeled using the OpenMM molecular dynamics package [55]. The chromosomes are represented as polymer chains of spherical subunits with a DNA content of 100 kb, chain lengths being 1818, 1526 and 908 respectively. The first 30 subunits (3 Mb) of each chain correspond to the centromeric region. The initial state of the chain is a self-avoiding polymer coil placed in an impenetrable spherical cage with a radius equal to 14 subunit diameters. The cage is incorporated to speed up the equilibration process. The system is equilibrated until the radius of gyration ceases to change. At t > 0, the interaction potentials are turned on and the cage is removed. The interaction potential between any pair of non-adjacent subunits (i,j) consists of two components, U_ij_(r) = U_ev_(r) + U_attr_(r,i,j), where U_ev_(r) is the excluded volume and U_attr_(r,i,j) is the attracting potential. Excluded volume is modeled by the Weeks-Chandler-Andersen potential with the same coefficient U_0ev_ for all subunit pairs:

(1) Uevr=4U0ev[(dr)12−(dr)6+14],  r≤d2160,  r>d216

where d is subunit diameter. The attracting potential is modeled by the shifted and truncated Lennard–Jones potential with the coefficient U_0attr_ depending on subunit positions along the chain:

(2) Uattrr,i,j=0,  r≤d2164U0attri,j[(dr)12−(dr)6−(drcut)12+((drcut)6],  d216<r≤rcut0,  r>rcut

where r_cut_ is the distance between the centers of the elements, from which the potential is not calculated. We use the value r_cut_ = 3d, at which the non-displaced value of the potential is 0.5% of the depth of the well and therefore we can neglect attraction at distances greater than r_cut_.

The coefficients for the attracting potentials are determined in such a way that the ensemble of polymer conformations reproduces the experimental Hi-C contact frequency map. For this purpose, the following iterative algorithm is used:

1. The initial coefficients are equal for all element pairs, U_0_ = 1.2 kT.
2. The ensemble of up to 4000 conformations is simulated.
3. The contact map with 100 kb resolution is obtained. Pearson’s correlation between the simulated map and the experimental one is calculated.
4. If Pearson’s correlation between the contact maps ceases to improve, the simulation is stopped. Otherwise, for each element pair (i,j), the coefficient in the attraction potential increases if the simulated contact frequency is lower than the experimental one and decreases if it is higher.
5. Return to step 2.

As a result, we obtain the potentials and the ensemble of 3D conformations of chromosomes optimized to the best description of the experimental Hi-C maps. Thus, the population-averaged contact map corresponds to the heterogeneous set of structures and not to one “average” conformation.

For kth chromosome conformation from the ensemble, the contact between loci i and j (or (i,j) contact), p^k^_cont_(i,j) is scored if distance between them (centers) not exceeding R_cont_:

(3) $$p_{\mathrm{cont}}^{k}\left(i,j\right)=\left\{\begin{array}{c} 1,\;\;r\left(i,j\right)\leq R_{\mathrm{cont}}\\ 0,\;\;r\left(i,j\right)>R_{\mathrm{cont}} \end{array}\right.$$

and we assume R_cont_ = 1.2 d. Contacts between all loci i and j in a given conformation represent the single-chromosome contact map.

The ensemble-averaged contact map (step 3) is the sum of the contacts between the loci i and j over the ensemble of chromosome conformations p_cont_(i,j)

(4) $$p_{\mathrm{cont}}\left(i,j\right)=\overset{K}{\underset{k=1}{\sum }}p_{\mathrm{cont}}^{k}\left(i,j\right)$$

where K is the number of chromosomes/conformations in the ensemble.

The number of contacts in the chromosome ensemble between loci i and j is normalized as

(5) $$C\left(i,j\right)=\frac{p_{\mathrm{cont}}\left(i,j\right)}{\sum_{\begin{array}{c} i,j\\ \left|i-j\right|>1 \end{array}}p_{\mathrm{cont}}\left(i,j\right)}$$

where the denominator is the total number of contacts of locus pairs in the ensemble of conformations. Contacts between neighbor elements (genomic separation s = 1) are excluded.

The single-cell contact map is defined as the sum of the contact maps for homolog chromosomes. To compare with experiments, the population-averaged contact map is defined as the sum of the single-cell contact maps divided by the number of cells in a cell population. The experimental Hi-C contact matrix with a given resolution is normalized also according to Equation (5) where the numerator is the number of counts between loci i and j in a cell population, the denominator is the total number of counts between all pairs of loci. s = 1 is excluded.

C(i,j) in Equation (5) is the relative number of contacts, or the relative contact frequency per chromosome. The absolute frequency of contact between loci i and j in the ensemble is defined as a fraction of chromosome conformations in which loci i and j are in contact. According to Equation (3) the number of conformations with (i,j) contacts, equals the number of (i,j) contacts in ensemble (Equation (4)). Then the absolute frequency of (i,j) contacts is defined as

(6) $$q_{\mathrm{cont}}\left(i,j\right)=\frac{p_{\mathrm{cont}}\left(i,j\right)}{K}.$$

The sense of q_cont_(i,j) is the chromosome ensemble-averaged value or the average number of contacts per chromosome. Since K = 2M, where M is the number of cells in a population, the absolute contact frequency in cell population between loci i and j equals

(7) $${\tilde{q}}_{\mathrm{cont}}\left(i,j\right)=\frac{p_{\mathrm{cont}}\left(i,j\right)}{M}=2q_{\mathrm{cont}}\left(i,j\right).$$

${\tilde{q}}_{\mathrm{cont}}\left(i,j\right)$ is the cell population-averaged value or average number of contacts per cell.

We normalize experimental contact maps according to Equation (5) to obtain relative frequencies. Using absolute frequencies for comparison with calculations is impossible since efficiency of contact detection in Hi-C experiments is unknown.

Contact frequency of two loci as a function of genomic separation s, f(s) is determined on the basis of C(i,j) from Equation (5) as

(8) $$f\left(s\right)=\overset{N-s}{\underset{i=1}{\sum }}C\left(i,i+s\right)$$

where N is the number of subunits in the polymer chain. The centromeric and non-mappable regions are excluded from summation.

The contacts with the centromeric monomers are taken into account during chromosome dynamics. To avoid misclassification during the comparison with experiment, contacts with centromeric subunits are not counted in steps 3 (contact map calculation and comparison) and 4 (modification of potentials), since the centromeric region is not assayed in Hi-C. Non-mappable regions (white strips on the experimental Hi-C maps) are accounted for as follows. Since there is no experimental basis for determining the structure of non-mappable regions, we postulate that it does not differ considerably from that of neighboring mappable regions. Therefore, the potentials for attraction between a non-mappable subunit i with any subunit j are taken as averaged over the mappable subunits closest to i. During comparison between the simulated and the experimental maps, non-mappable subunits are excluded, since the experimental data on contacts are absent. Since the model considers individual chromosomes, it does not take into account interactions with other chromosomes as well as with other nuclear structures (nucleolus, lamina, etc.).

To explore how spatial heterogeneity of chromosome structure in a cell population impacts the inhomogeneous breakpoint distribution along the chromosome, we determine distributions of 3D distance between chromosome loci in the chromosome conformation ensemble, functions ψ_i,j_(r) and Φ_i,j_(r;D) for all and damaged loci (at dose D), respectively. The function ψ_i,j_(r)d^3^r is the number of locus pairs (i,j) in the ensemble of chromosome conformations with the 3D distance r, r + dr, located in the spherical layer d^3^r.

The function ψ_i,j_(r) is obtained from an ensemble of 3D conformations. The statistical ensemble of conformations is described in full by a many-particle distribution function of the coordinates of all loci in the polymer which is obtained from the molecular dynamics simulations. Therefore, the physical sense of ψ_i,j_(r) is a pair correlation function which is calculated as an integral of the many-particle distribution over coordinates of other loci in the chromosome, provided that 3D distance between loci i and j remains r, r + dr. The distance distributions per chromosome ψ_i,j_(r) and φ_i,j_(r;D) are obtained from distributions for ensemble ψ_i,j_(r) and Φ_i,j_(r;D) as

(9) $$\psi_{\mathrm{ij}}\left(r\right)=\frac{\Psi_{\mathrm{ij}}\left(r\right)}{K},\;\;\phi_{\mathrm{ij}}\left(r\right)=\frac{\Phi_{\mathrm{ij}}\left(r\right)}{K}.$$

The relationship between per-chromosome functions φ_i,j_(r;D) and ψ_i,j_(r) is defined by the equation

(10) $$\phi_{i,j}\left(r;D\right)=\psi_{\mathrm{ij}}\left(r\right)\left[P_{\mathrm{DSB}}\left(1,j;D\right)+\Gamma_{\mathrm{sp}}\left(1,j\right)\right]$$

where P_DSB_ (1,j;D) is a probability of one DSB induced by IR in locus j, jth subunit of a polymer chromosome. Γ_sp_(1,j) is the distribution of spontaneous DSBs in locus j along the chromosome. Recurrent (I-SceI nuclease-induced) DSBs are simulated as present in I-SceI recognition site in locus i with probability 1. From the Poisson distribution of the number of DSBs n, P(n,$\bar{n}$), for low mean $\bar{n}$ << 1, P(1,$\bar{n}$) ≈ $\bar{n}$. For a 100 kb subunit, $\bar{n}$ = α·D·L, L = 10^5^ bp, α is efficiency of DSB induction, for γ-radiation we take α = 8.2·10^−9^ Gy^−1^ bp^−1^ [56], hence $\bar{n}$ = 8.2·10^−4^D for any j. Then $\bar{n}$ << 1 for all doses D ≤ 15 Gy, and P_DSB_(1,j;D) ≈ α·D·L. Function of distance distribution per cell is determined as 2φ_i,j_(r;D).

### 4.2. Simulation of Trial Structures

A number of trial structures used in CA modeling was simulated for comparison with experimental data and the heteropolymer chromosome model: a homopolymer globule (for chromosome 2 U_0attr_ = 1.57 kT, for chromosome 7 U_0attr_ = 2.33 kT, for chromosome 18 U_0attr_ = 9.34 kT), a loose homopolymer globule (only for chromosome 18, U_0attr_ = 1.2 kT), a self-avoiding polymer coil with the excluded volume interactions (U_0attr_ = 0), RW or a Gaussian model without excluded volume interactions [26] (U_0attr_ = U_0ev_ = 0). The macroscopic characteristics of homo- and heteropolymer structures obtained in this work are shown in Figures S1 and S2.

### 4.3. Modeling of Intrachromosomal Aberrations by the Contact-First Mechanism

The distribution of chromatin damage in chromosomes is simulated by the Monte Carlo technique as follows. One chromosome conformation from an optimized ensemble of structures characterized by a certain pattern of contacts is randomly selected. The number of IR-induced DSBs in the conformation is sampled from the Poisson distribution and the location of DSBs is stored. The number of spontaneous DSBs is generated by another algorithm described below. The recurrent DSB is considered as present in locus i, nuclease recognition site. A subunit where one of three types of DSBs occurs, radiation-induced, spontaneous or nuclease-induced, is termed as a damaged subunit. By contact of damaged subunits, we mean contact of any subunits i and j, provided that both of them carry DSBs. When a contact of damaged subunits for the given chromosome conformation is determined, it is converted into an exchange aberration with the probability of P_c-e_ [57], and the position of exchange between elements i and j, or aberration breakpoint position, is stored. Symmetrical (inversions) and asymmetrical (rings) exchanges are formed with equal probabilities. All previous steps are repeated twice independently for two chromosomes. For a given cell, the two distributions are summed up, so that the single-cell distribution of translocation breakpoints is obtained. This is the single-cell contact approach to the determination of translocation breakpoint distributions.

Since for comparison with experiments simulations are needed for a huge number of cells or conformations (millions), we made the following approximation. For each contact pattern, induction of DSBs is sampled independently many times (10^5^), the frequency of DSBs in each locus is determined, then averaged over the number of samples (per conformation) and then over the number of conformations. Our approximation is that the number of conformations and the number of samples with radiation damage were different, although they should be the same. The estimates show that this approximation barely affects the results for a population-averaged distribution of lesion contacts and a sufficient number of conformations.

For comparison with experimental population-averaged Hi-C and HTGTS quantities, the number of cells simulated should be very large (millions). To avoid some uncertainties related to the experimental design and to compare with experiments, we determined the “population-averaged” distribution, averaged over “infinite” (very large) number of cells, and the distribution, averaged over the finite number or pool of cells in the population. The “infinite” population of cells is defined in such a way that the distribution shape is not changed with increasing the number of cells. In our case, we averaged the single-cell breakpoint distribution over M~10^8^ cells. The simulation of a larger number of cells is superfluous. The distributions averaged over a pool of cells (M~10^4^–10^7^) are obtained similarly and are sensitive to the number of cells.

The relationship between population-averaged (f_bp_) and single-chromosome (f^k^_bp_) breakpoint distributions is described by the following equation:

(11) $$f_{\mathrm{bp}}\left(i,j;D\right)=\frac{2}{K}\overset{K}{\underset{k=1}{\sum }}f_{\mathrm{bp}}^{k}\left(i,j;D\right)=\frac{2}{K}\overset{K}{\underset{k=1}{\sum }}P_{\text{c-e}}p_{\mathrm{cont}}^{k}\left(i,j\right)\left[P_{\mathrm{DSB}}^{k}\left(1,j;D\right)+\Gamma_{\mathrm{sp}}^{k}\left(1,j\right)\right]$$

where f_bp_(i,j;D) is the number of breakpoints per cell averaged over the population of M = K/2 cells. Superscript k denotes kth chromosome, p^k^_cont_(i,j) is the number of contacts between loci i and j in kth chromosome (Equation (3)).

As an alternative to the breakpoint calculations through single-cell contacts (Equation (11)), we employ another method, based on the distance distributions between lesions, thus taking spatial heterogeneity function φ_i,j_(r;D) into account explicitly. The number of contacts per cell between the recurrent DSB induction site i and the lesion in locus j is determined as

(12) $$f_{\mathrm{cont}}\left(i,j;D\right)=2\int_{0}^{R_{\mathrm{cont},\mathrm{CA}}}\phi_{i,j}\left(r;D\right)4{\pi r}^{2}\mathrm{dr}$$

where R_cont,CA_ is the size of lesion colocalization area necessary for CA formation. Here we take R_cont,CA_ = 1.2d, i.e., the same as R_cont_ for structure simulation, though in the general case they are different. According to the contact-first mechanism, every contact gives rise to exchange with probability P_c-e_, and the frequency of breakpoints in the subunit j f_bp_(i,j;D) is obtained as

(13) $$f_{\mathrm{bp}}\left(i,j;D\right)=f_{\mathrm{cont}}\left(i,j;D\right)P_{\text{c-e}}.$$

Equation (13) is used for assessment of P_c-e_ from the best agreement (minimal Chi-square) between the simulated and the experimental distributions. Chi-square is calculated as follows:

(14) $$\chi^{2}=\underset{j}{\sum }\frac{{\left(f_{\mathrm{bp}}^{\mathrm{sim}}\left(j;D\right)-f_{\mathrm{bp}}^{\exp}\left(j;D\right)\right)}^{2}}{f_{\mathrm{bp}}^{\mathrm{sim}}\left(j;D\right)}$$

where superscripts sim and exp mean simulated and experimental values, respectively. The resulting P_c-e_ values are: 0.0035 for chromosome 2; 0.0029 for chromosomes 7 and 18. In a general case, this value can depend on locus position on the chromosome, but in the present work, this possibility is not explored. The repair process in ATM-deficient cells is not incorporated explicitly; implicitly it is manifested in P_c-e_ values. This means that wild-type cells could have P_c-e_ values different from what we obtained for repair-deficient cells.

### 4.4. Aberration Modeling in the Absence of Irradiation

For modeling of CAs formed in the absence of irradiation (D = 0), one needs to determine the distribution of spontaneous DSBs in locus j along the chromosome, Γ_sp_(1,j). This information is not available in Experiment [21], unlike the spontaneous aberration breakpoint distribution. To reconstruct the missing information about DSBs, we suggest the following method. The experimental data on spontaneous breakpoints are available with different bining: 2 Mb on the level of the entire chromosome, and 25 kb in range 69.6–72.0 Mb only for chromosome 18. The breakpoint distribution is required with 100 kb bining since DNA content of a polymer subunit in the structure model is 100 kb that corresponds to the highest resolution Hi-C contact map for mouse chromosomes in [21]. To obtain the spontaneous breakpoint distribution with 100 kb, the distribution of spontaneous breakpoints with 2Mb resolution is interpolated by Akima spline with a 100 kb bin. Following interpolation, the breakpoint distribution is normalized to the experimental one requiring the equality of total number of breakpoints. In the range 69.6–72.0 Mbp the interpolated breakpoint distribution for chromosome 18 is replaced with the experimentally known high (25 kb) resolution distribution. So, we obtain 100 kb distribution of spontaneous breakpoints for the entire chromosome 18.

From the spontaneous breakpoint distribution, we extract missing information about the distribution of spontaneous DSBs with 100 kb resolution. We exploit the following iterative algorithm. First, for the initial uniform DSB distribution, the breakpoint distribution is simulated for the heteropolymer globule model of the chromosome and for the contact-first mechanism. Second, for loci with the calculated breakpoint frequency lower than the experimental one, spontaneous DSB induction is increased, and vice versa. Third, the modified DSB distribution is applied to calculate the next iteration of the breakpoint distribution, and so on. It is repeated until Pearson’s correlation between the simulation and the experiment ceases to improve. The resulting DSB and breakpoint distributions for chromosome 18 are shown in Figure S16. Spontaneous DSBs for chromosomes 2 and 7 are not simulated, since the data [21] demonstrate that under 5 Gy irradiation the contribution of spontaneous breakpoints to the total frequencies is negligible for all chromosomal loci.

### 4.5. CA Modeling by the Breakage-First Mechanism

The initial state of the system is the ensemble of equilibrated conformations describing the contact map. One conformation is chosen randomly, and DSB (IR-induced, spontaneous, recurrent) distribution of damaged loci along the chromosome, is simulated in the same way as for the contact-first mechanism. Next, the dynamics of lesions in the polymer chain in the form of 3D-and-time trajectories are tracked. We suggest that the dynamics of lesions following irradiation are the same as loci dynamics in nonirradiated cells. This suggestion correlates with data [42].

The dynamics of lesion contact formation are simulated in the following way. First, the function *ρ*_ij_(r_0_,r,t;D) is introduced. This function is density of lesion j in the spherical layer (r, r + dr) from lesion i at moment t, under condition that at t = 0, the distance was (r_0_, r_0_ + dr). This function enables determining the frequency of contacts formed within any time range (Figure S13) as well as in the entire time range from 0 to t (Figure 8). Next, on the basis of these quantities, CAs are calculated in the following way. The parameter which governs lesion interaction process is contact-exchange rate V_c-e_ rather than probability P_c-e_ which was used for the contact-first mechanism. If two damaged elements come into contact, the formation of an exchange aberration occurs with probability V_c-e_·δt where δt is a time step in the dynamic model. If the lesions are in contact initially, then CA is also formed with probability V_c-e_·δt. This means that the calculations include the contact-first mechanism too, as an integral part. Since CAs are studied in cells mutated by the ATM repair gene, we suggest that the number of lesions is not decreased during movement until lesion contact formation. We neglect also the possibility that the lesion contact which did not lead to CA formation could decay and form again after some time.

In molecular dynamics simulations, we use the internal size and time scales which later are converted to real ones. The internal parameters were taken from software using the same OpenMM package [58,59]. The main parameters are: subunit mass 100 amu, d = 1 nm. According to [60], the characteristic Lennard–Jones time τ = d(m/U_0_)^1/2^, and friction coefficient Γ = 0.5/τ, which, for U_0_~1 kT, gives estimates τ ≈ 6 ps and Γ ≈ 0.1 ps^−1^.

Spatial and temporal scales used in our molecular dynamics calculations are converted to the real scales by using the data on mobility of IR-induced DSB containing chromatin loci, 53BP1 foci [39]. The comparison between the simulated mean squared displacement for several damaged loci of chromosome 18 (heteropolymer globule) and the experimental data [42] is presented in Figure S12. We use diameter 225 nm and the conversion “1 real hour = 5500 ps of simulation” as an estimate for the calculations. The time range of our CA simulations is 18,000 ps which corresponds to 3.3 h. The elementary time step for contact formation calculations δt equals 5 ps in molecular dynamics simulations which corresponds to 3.3 s of real time.

The experiments [42] on DSB containing locus dynamics used here for conversion of spatial and temporal scales of the model were carried out for the cell lines other that are studied here and in [21]. This is the potential source of uncertainty in rescaling.

### 4.6. CA Modeling Incorporating Conformational Transitions

The hypothetical conformational change of chromosome structure, i.e., a non-diffusive displacement of loci is introduced through the alteration of volume interaction potentials. The conformational transitions following irradiation are incorporated into the model by means of two scenarios.

In the first scenario, instead of a series of conformational transitions, we divide the entire time range into two intervals, provisionally “early” and “late” times. It is assumed that a conformational transition occurs after a long time post-irradiation. “Early” time corresponds to the initial set of potentials which described Hi-C data. CAs on early time scale are formed by the contact-first mechanism. “Late” time corresponds to the altered set of potentials and the conformational transition. After the change of structure, CAs are formed by the contact-first mechanism, similar to “early” time. The total frequency of breakpoints is determined as the sum of those formed at early and late times. The relative weights of early vs. late times are introduced as free parameters and are determined by the best fit (Chi-square minimization) of the calculated and experimental breakpoint distribution. This scenario was tested on all three studied chromosomes, using the chromosome model as a heteropolymer globule.

In the second scenario, the first part of CAs is formed at t = 0 by the contact-first mechanism. Then the conformation transition occurs. After that, lesion dynamics start in the altered structure of the chromosome, and formation of aberrations at later times (t > 0) by the breakage-first mechanism takes place. The aberrations are counted at the time of observation as a sum at t = 0 and t > 0. This scenario was implemented only for chromosome 18.

One can note the uncertainties and restrictions of both scenarios. For the mouse cell line considered, there are no Hi-C experiments after γ-irradiation. Therefore, the independent determination of the potentials after conformational transition is impossible. The data on breakpoint frequencies are available only for CAs induced by IR and targeted DSBs with the I-SceI nuclease. Based on these data, we were able to predict changes of potentials U_0attr_ only for the fixed i corresponding to the I-SceI recognition site. All other potentials in our calculations were assumed unchanged. Thus, we do not claim that the models of structure changes under conformational transitions obtained in the present work are the only possible ones. They are just a sample from many possible variants. Conformational transitions occur in the model locally rather than chromosome-wide and are not visible on the level of a whole contact map.

Comparison between Chi-square values obtained for different mechanisms (conformational transitions (Figure S15); contact-first (Figure 3); contact-first and breakage-first (Figure 9)) showed that the conformational transitions improved the joint description of simulated and experimentally observed breakpoint distributions significantly, from 40–90 (contact-first) and 30–75 (contact-first and breakage-first) to ~8 (conformational transitions) for all three chromosomes studied.

### 4.7. Data Analysis

Structural data. Hi-C maps for G1-arrested A-MuLV-transformed ATM^−/−^ mouse pro-B cells were taken from GEO (accession number GSE35519) referred to in [21]. For quantitative comparison of the calculated and experimental data on the structure of mouse chromosomes [21], all maps were normalized to the total number of contacts at genomic separations greater than 100 kb, i.e., size of a polymer subunit and size of a bin that we chose for analysis of experimental heatmaps. The experimental maps have been corrected for biases according to the coverage of each bin in [21]. The Pearson’s correlation was used as a criterion for the agreement between the calculation and the experiment. For comparison with the data on breakpoints involving I-SceI-induced DNA DSBs, only contacts with subunits containing the I-SceI recognition site were used. In chromosome 2, this is the subunit 180.1–180.2 Mb, in chromosome 7, the subunit 31.2–31.3 Mb, in chromosome 18, the subunit 70.6–70.7 Mb. These subsets of Hi-C data are equivalent to the data of 4C experiments with the bait (pseudo-4C) at the corresponding loci.

Breakpoint distribution data. Since the database in GEO referred to in [21] does not contain breakpoint data, we extracted them directly from the graphs in the paper. The simulated distributions underwent the same procedure as the experimental ones. They were enlarged to a resolution of 200 kb and smoothed with a sliding window of 1 Mb. For region 69.6–72.0 Mb in chromosome 18, smoothing was not done, since the experimental data for this region are available with 25 kb resolution. The shape of the distributions was compared using the Pearson’s correlation, while, as in [21], loci in the 1 Mb region around the I-SceI recognition site were not taken into account.

For quantitative comparison between the simulated and the experimental breakpoint distributions, the same locus range is required. For chromosomes 2 and 7 and 200 kb, resolution the area near the peak on the graphs (Figure 6 and Figure S6 in [21]) that we digitized were uncertain due to the peak’s truncation. For this reason, we did not take into account the missing areas. The excluded uncertain area was 179.6–181.2 Mb for chromosome 2 and 31.0–32.2 Mb for chromosome 7. There is no such problem for chromosome 18.

## Figures and Tables

**Table 1:** Relative fluctuations of characteristics for chromosome 18.

Characteristics	Relative Fluctuations, SD/Mean
Number of total contacts in chromosome	0.014
Number of contacts with I-SceI site	0.33
Number of lesion contacts with I-SceI site (5 Gy)	4.32
Number of breakpoints (5 Gy)	110.6

## Data Availability

The experimental Hi-C data on the structure of mouse chromosomes 2, 7, 18 [21], which were analyzed here, are available on Gene Expression Omnibus (GEO), accession number GSE35519. The simulation data on ensembles of 3D conformations of mouse chromosome, the damage heterogeneity function as well as the time-dependent contact formation functions are posted on the website AndreevLab. Available online: https://andreevlab.biochemphysics.ru/resources/simulation-data/chromosome-folding-promotes-intrachromosomal-aberrations/ (accessed on 5 November 2021). The following datasets are available: *Conformation ensembles*: Chromosome 18, heteropolymer globule; Chromosome 18, homopolymer globule; Chromosome 18, loose globule; Chromosome 18, coil; Chromosome 18, heteropolymer globule after conformational transition at late time; Chromosome 18, heteropolymer globule after conformational transition at early time; Chromosome 7, heteropolymer globule; Chromosome 7, homopolymer globule; Chromosome 7, coil; Chromosome 7, heteropolymer globule after conformational transition at late time; Chromosome 2, heteropolymer globule; Chromosome 2, homopolymer globule; Chromosome 2, coil; Chromosome 2, heteropolymer globule after conformational transition at late time. *Damage heterogeneity function*: Chromosome 18, heteropolymer globule (i = 706, D = 0.1 Gy); Chromosome 18, heteropolymer globule (i = 706, D = 5 Gy); Chromosome 18, heteropolymer globule (i = 706, D = 15 Gy); Chromosome 7, heteropolymer globule (i = 312, D = 5 Gy); Chromosome 2, heteropolymer globule (i = 1801, D = 5 Gy). *Lesion contact formation function*: Chromosome 18, heteropolymer globule (i = 706, D = 5 Gy), differential; Chromosome 18, heteropolymer globule (i = 706, D = 5 Gy), integrated over time; Chromosome 7, heteropolymer globule (i = 312, D = 5 Gy), differential; Chromosome 7, heteropolymer globule (i = 312, D = 5 Gy), integrated over time; Chromosome 2, heteropolymer globule (i = 1801, D = 5 Gy), differential; Chromosome 2, heteropolymer globule (i = 1801, D = 5 Gy), integrated over time.

## Supplementary Materials

The following are available online at https://www.mdpi.com/1422-0067/22/22/12186/s1.
