# Supplementary material for: Chromosome Folding Promotes Intrachromosomal Aberrations under Radiation- and Nuclease-Induced DNA Breakage
Source: Int J Mol Sci. 2021 Nov 10;22(22):12186. doi: 10.3390/ijms222212186 (PMC8618582; doi:10.3390/ijms222212186)
Supplement: Supplementary file 1 [file ijms-22-12186-s001.zip › ijms-1430205-supplementary.pdf]

Chromosome Folding Promotes Intrachromosomal Aberrations Under Radiation- and Nuclease-Induced DNA Breakage

Eidelman Y.A., Salnikov I.V., Slanina S.V., Andreev S.G.

Supplementary materials

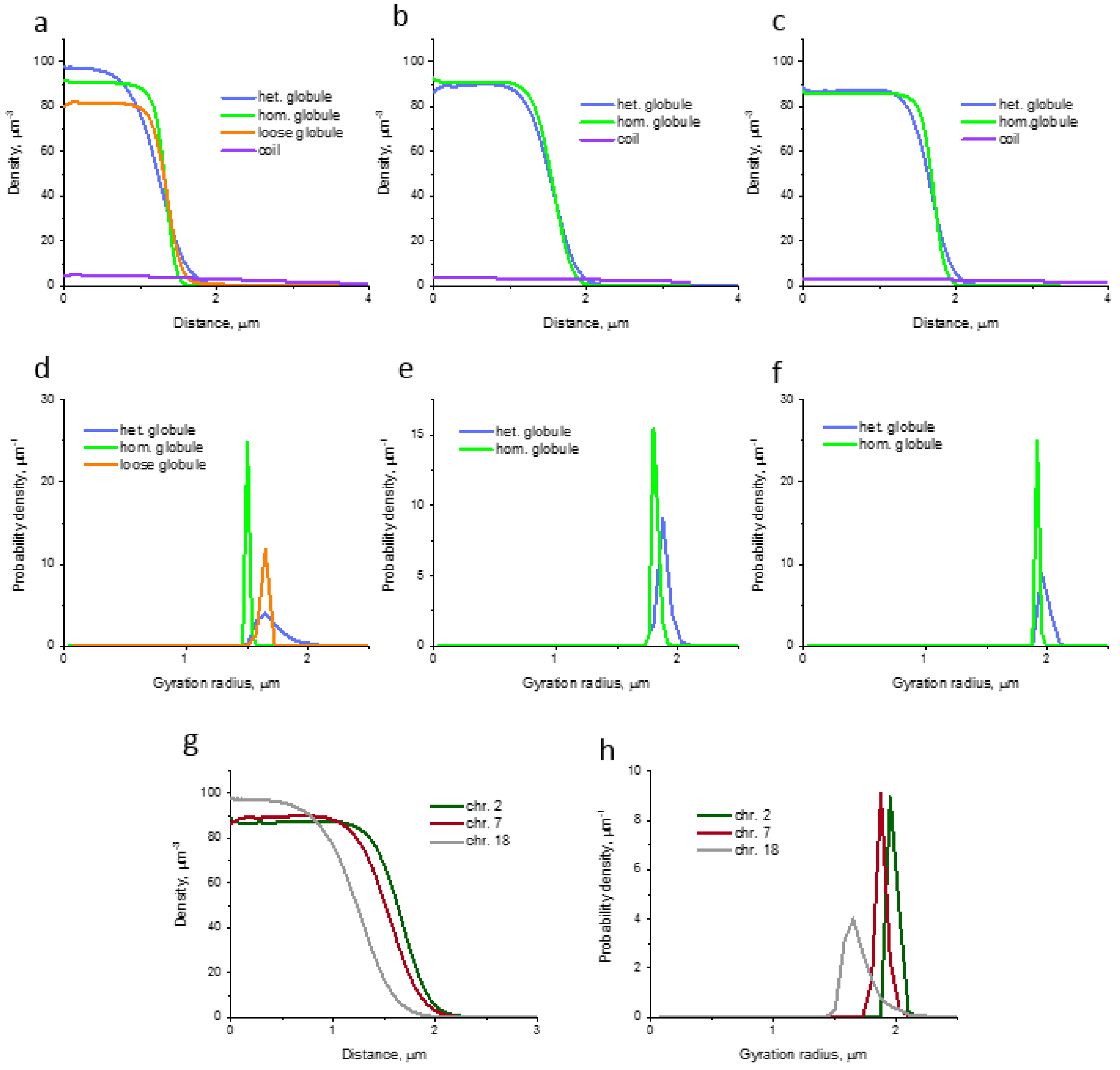

Figure S1. Structural characteristics of the models used. Models: heteropolymer globule (abbreviated as het. globule), homopolymer globule (hom. globule), loose globule (only for chromosome 18), self-avoiding polymer coil (coil). (**a** – **c**) radial density distribution. (**a**) Chromosome 18; (**b**) chromosome 7; (**c**) chromosome 2. (**d** – **f**) Gyration radius distribution. The same models except coil. (**d**) Chromosome 18; (**e**) chromosome 7; (**f**) chromosome 2. (**g**) Comparison between radial density distributions for chromosomes 18, 7, 2 as heteropolymer globules. (**h**) Comparison between gyration radius distributions for chromosomes 18, 7, 2 as heteropolymer globules.

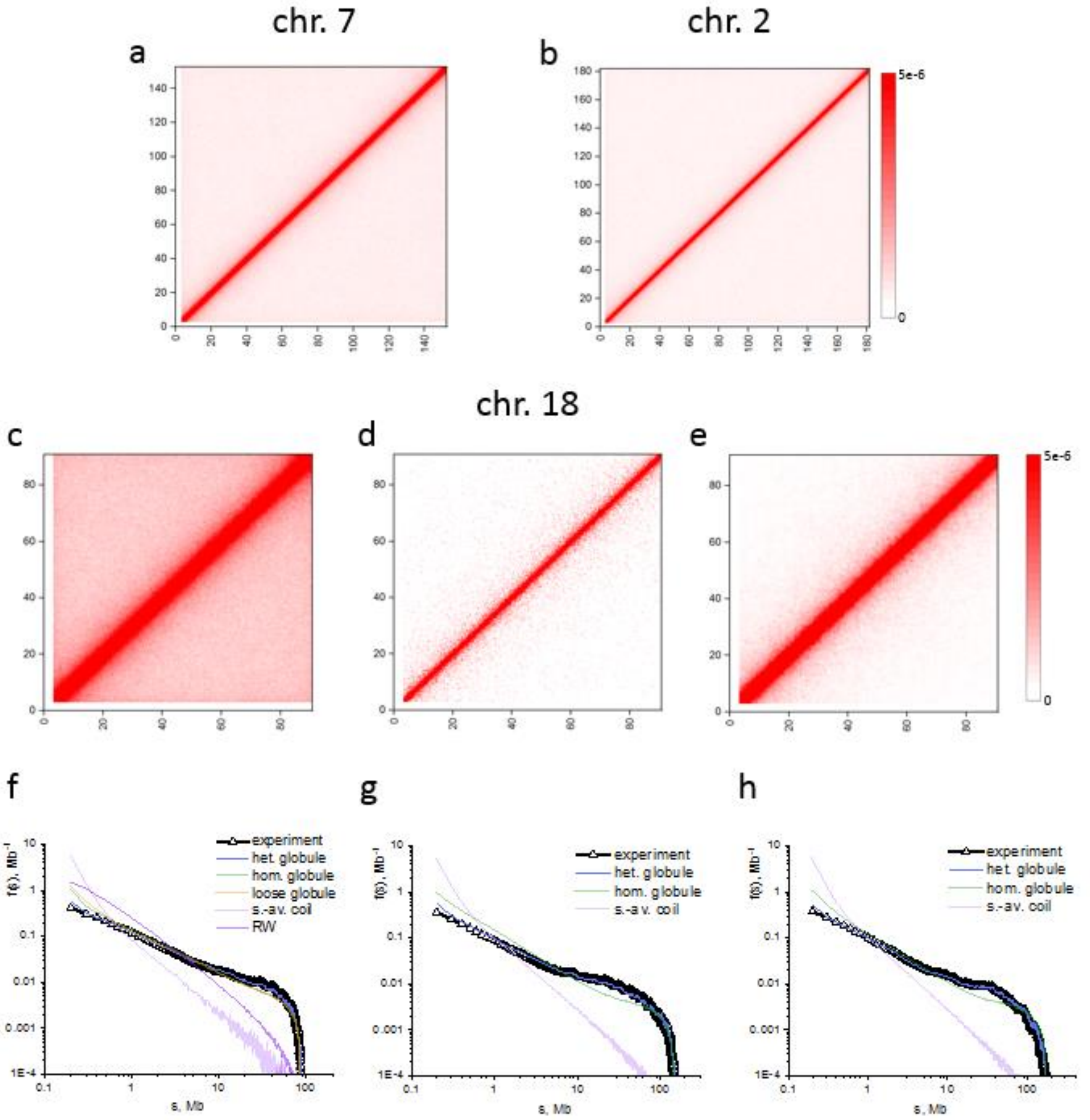

Figure S2. Structural characteristics of trial models of chromosomes 18, 7, 2. (**a** – **e**) contact maps. (**a** – **c**) homopolymer globules: (**a**) chromosome 7, (**b**) chromosome 2, (**c**) chromosome 18. (**d**, **e**) Coil models for chromosome 18. (**d**) Self-avoiding coil, (**e**) RW, ideal (Gaussian) coil. (**f** – **h**) Contact frequency as a function of genomic separation s, experiment [21] vs simulation for different chromosome models. (**f**) Chromosome 18; (**g**) chromosome 7; (**h**) chromosome 2.

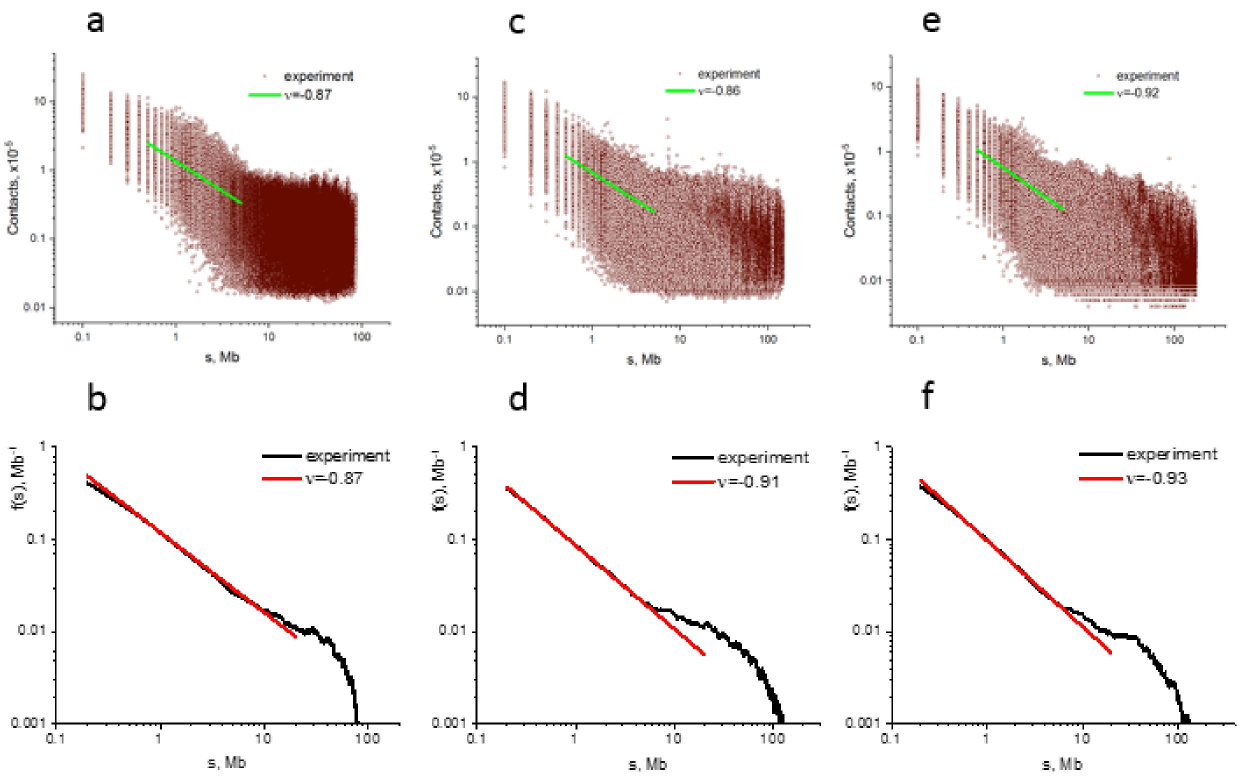

Figure S3. Power law fitting of contact-vs-genomic separation functions for the globular models of mouse chromosomes. (**a**, **b**) Chromosome 18; (**c**, **d**) chromosome 7; (**e**, **f**) chromosome 2. (**a**, **c**, **e**) The frequency of Hi-C contacts between loci in a chromosome as a function of genomic separation s. Each point is an individual pair of loci. The green line shows the approximation s^{ν} in the range of genomic separations 0.5 – 5 Mb. (**b**, **d**, **f**) Contact frequency as a function of genomic separation, the same as in Figure 2. The red line shows the approximation s^{ν} in the range of genomic separations 0.5 – 5 Mb. In all panels, the experimental data are derived from [21].

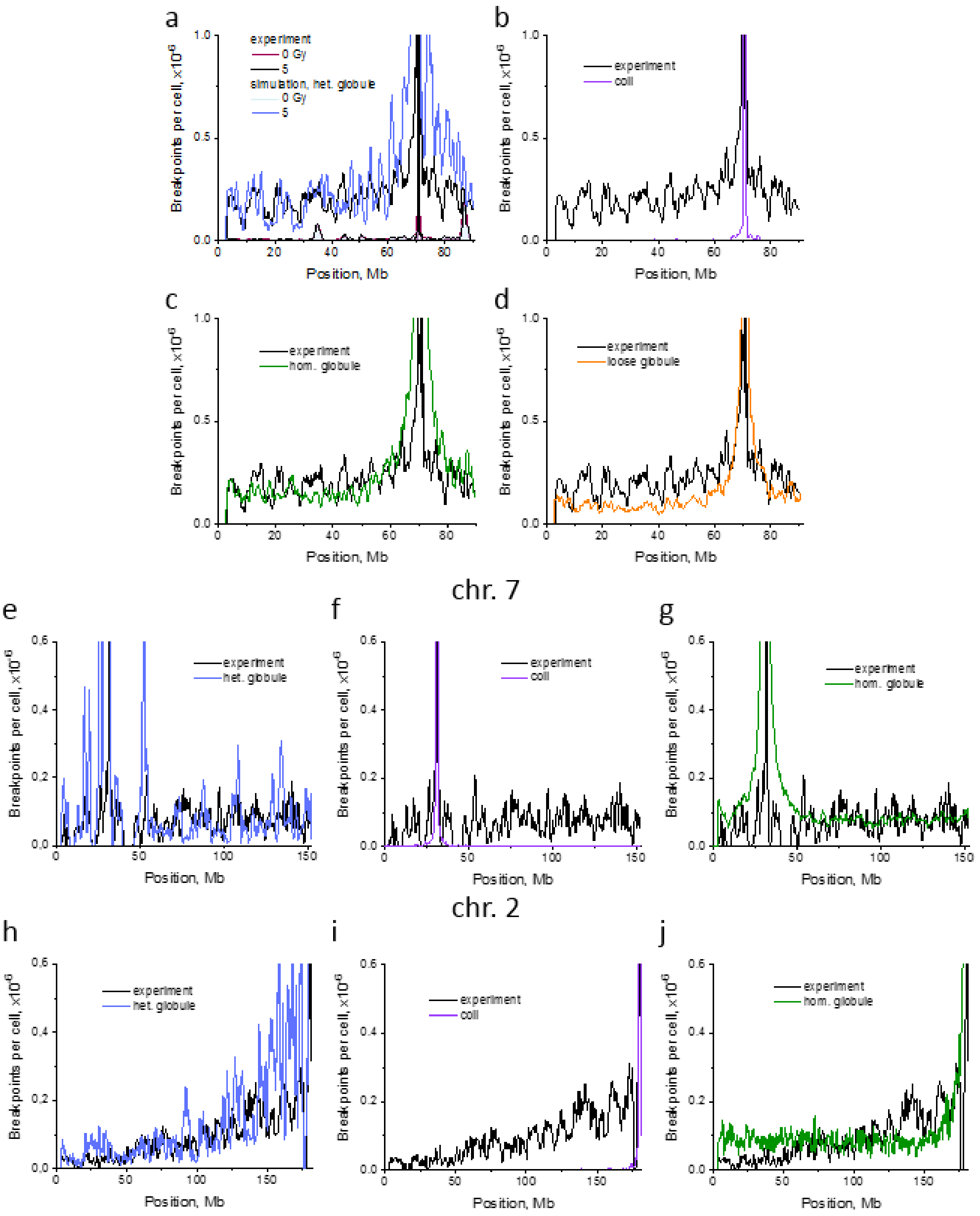

Figure S4. Cis-translocation breakpoint distributions in G1-arrested pro-B mouse cells evaluated by the contact-first mechanism and different structural models of chromosomes. In all panels: experiment [21]. (**a** – **d**) Chromosome 18, the probability of contact-exchange $P_{c\text{-}e}$=0.0029. (**a**) Calculations of translocation breakpoints based on a heteropolymer globular model (Figure 2 a). For radiation doses of 0 and 5 Gy, Pearson's correlation between theory and experiment R=0.898 and R=0.724, respectively. Following irradiation, the formation of translocations was taken into account both between recurrent and spontaneous, and between recurrent and IR-damaged chromosome subunits. (**b** – **d**) homopolymer models of structure of the chromosome 18 provide different predictions for the breakpoints in comparison with experimental data, D=5 Gy. (**b**) Prediction for the chromosome in the form of a self-avoiding polymer coil, R=0.586. (**c**) Homopolymer globule, R=0.740. (**d**) Loose globule, R=0.720. (**e** – **g**) Chromosome 7, D=5 Gy, $P_{c\text{-}e}$=0.0029. (**e**) Heteropolymer globule (Figure 2 e), R=0.299. (**f**) Polymer coil, R=0.293. (**g**) Homopolymer globule, R=0.335. (**h** – **j**) Chromosome 2, D=5 Gy, $P_{c\text{-}e}$=0.0035. (**h**) Heteropolymer globule (Figure 2 i), R=0.648. (**i**) Polymer coil, R=0.299. (**j**) Homopolymer globule, R=0.458. For chromosomes 2 and 7, the control in [21] is negligible compared to 5 Gy, so it is not shown in the graphs.

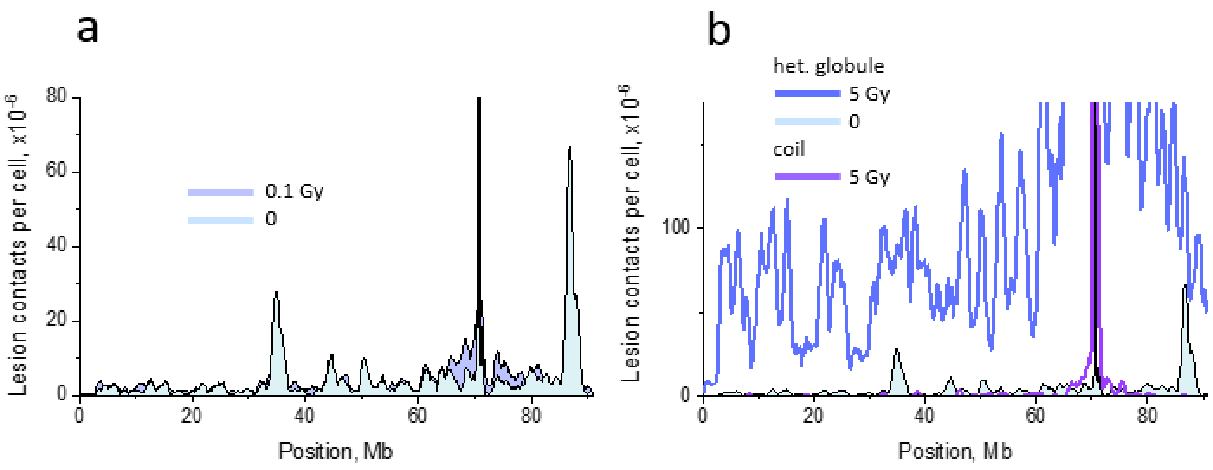

Figure S5. The contribution of IR-induced DSBs, spontaneous DSBs and the structural organization of the chromosome on the distribution of lesion contacts. Chromosome 18. (**a**) Heteropolymer globule model, doses 0 and 0.1 Gy. (**b**) Heteropolymer globule in comparison with the coil model, doses 0 and 5 Gy.

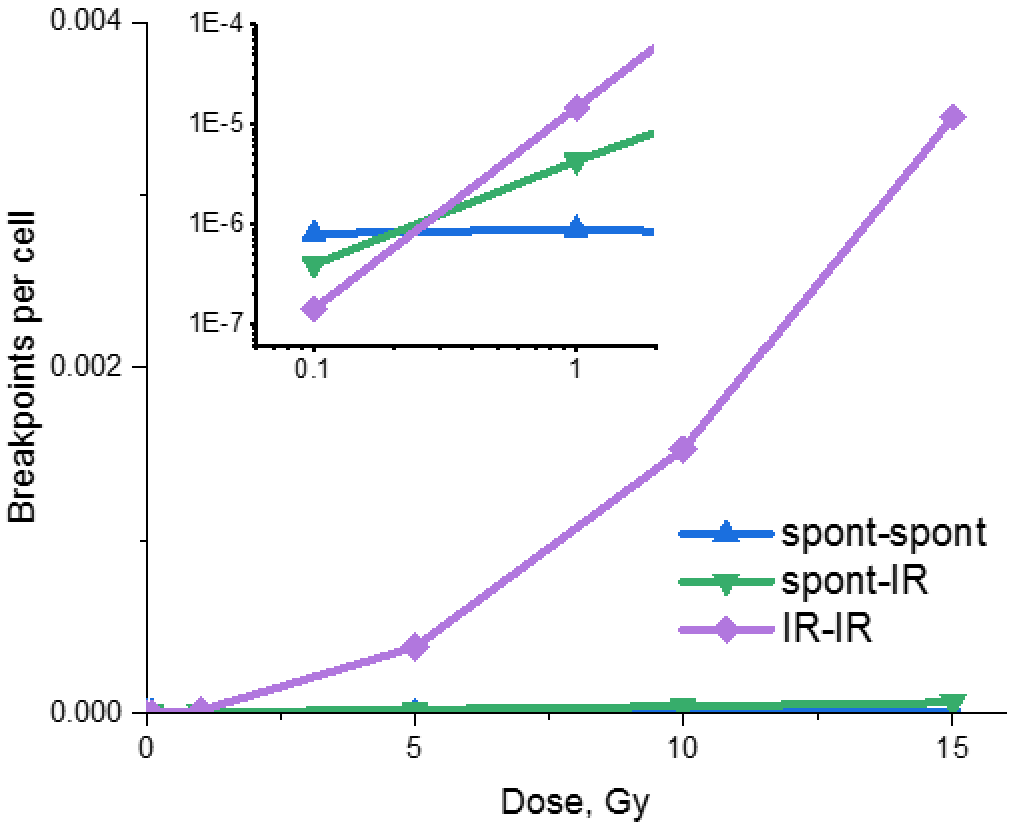

Figure S6. Dose dependence for the frequency of aberrations formed by lesions of different origins. Chromosome 18, heteropolymer model. Abbreviations: spont, spontaneous lesion; IR, IR-induced lesions. The inset shows the low-dose range.

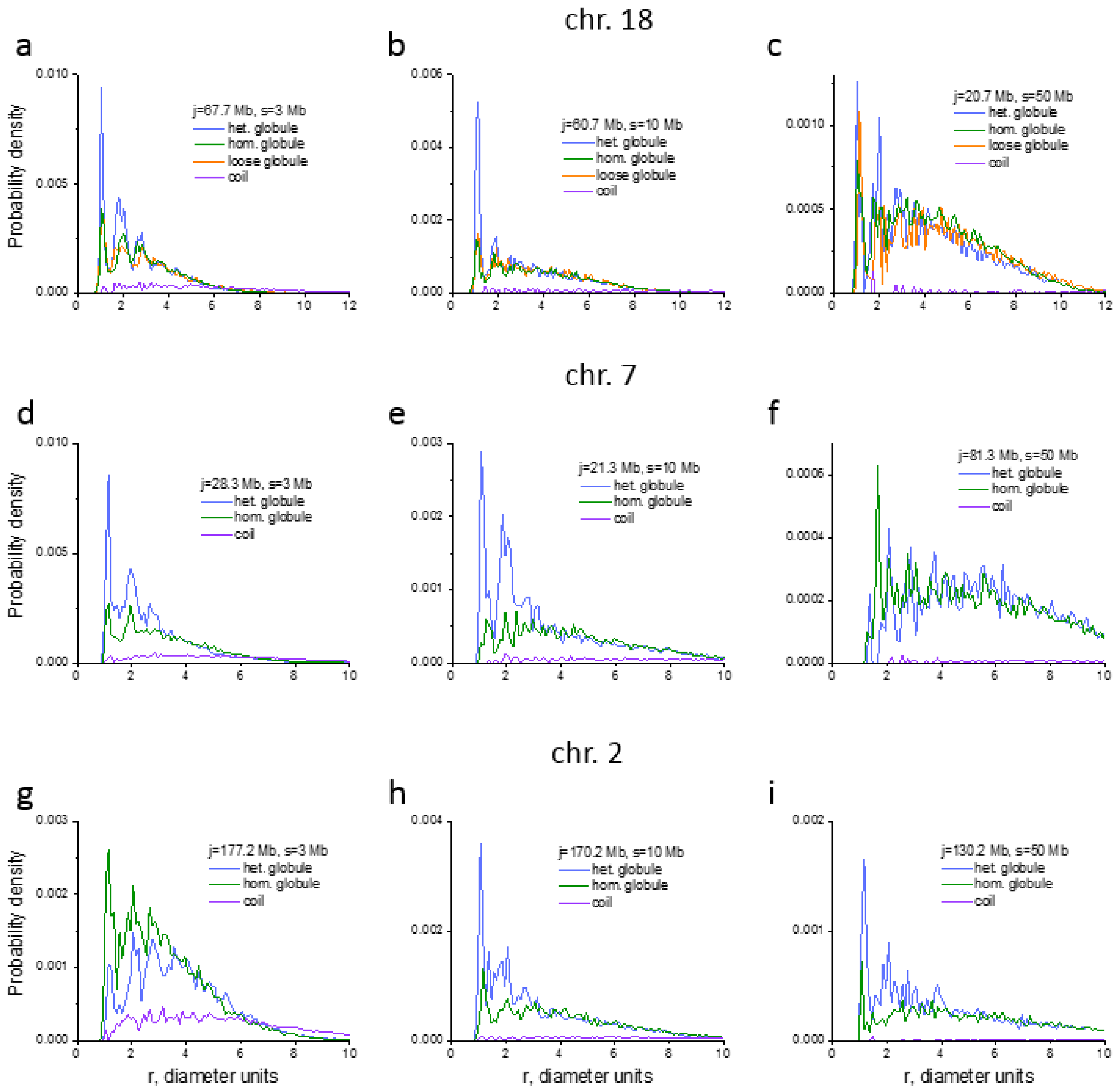

Figure S7. Distance distributions between loci i and j in chromosomes 2, 7 and 18, function $\psi_{i,j}(r)$. Locus i corresponds to the I-SceI site. Locus j position is shown in the panels. The calculations were performed for various structural states of chromosomes. Structure models: heteropolymer globule (abbreviated as het. globule), homopolymer globule (hom. globule), loose globule (only for chromosome 18), self-avoiding polymer coil (coil). (**a** – **c**) Chromosome 18; (**d** – **f**) chromosome 7; (**g** – **i**) chromosome 2). Genomic separations from the I-SceI site: (**a**, **d**, **g**) 3 Mb; (**b**, **e**, **h**) 10 Mb; (**c**, **f**, **i**) 50 Mb.

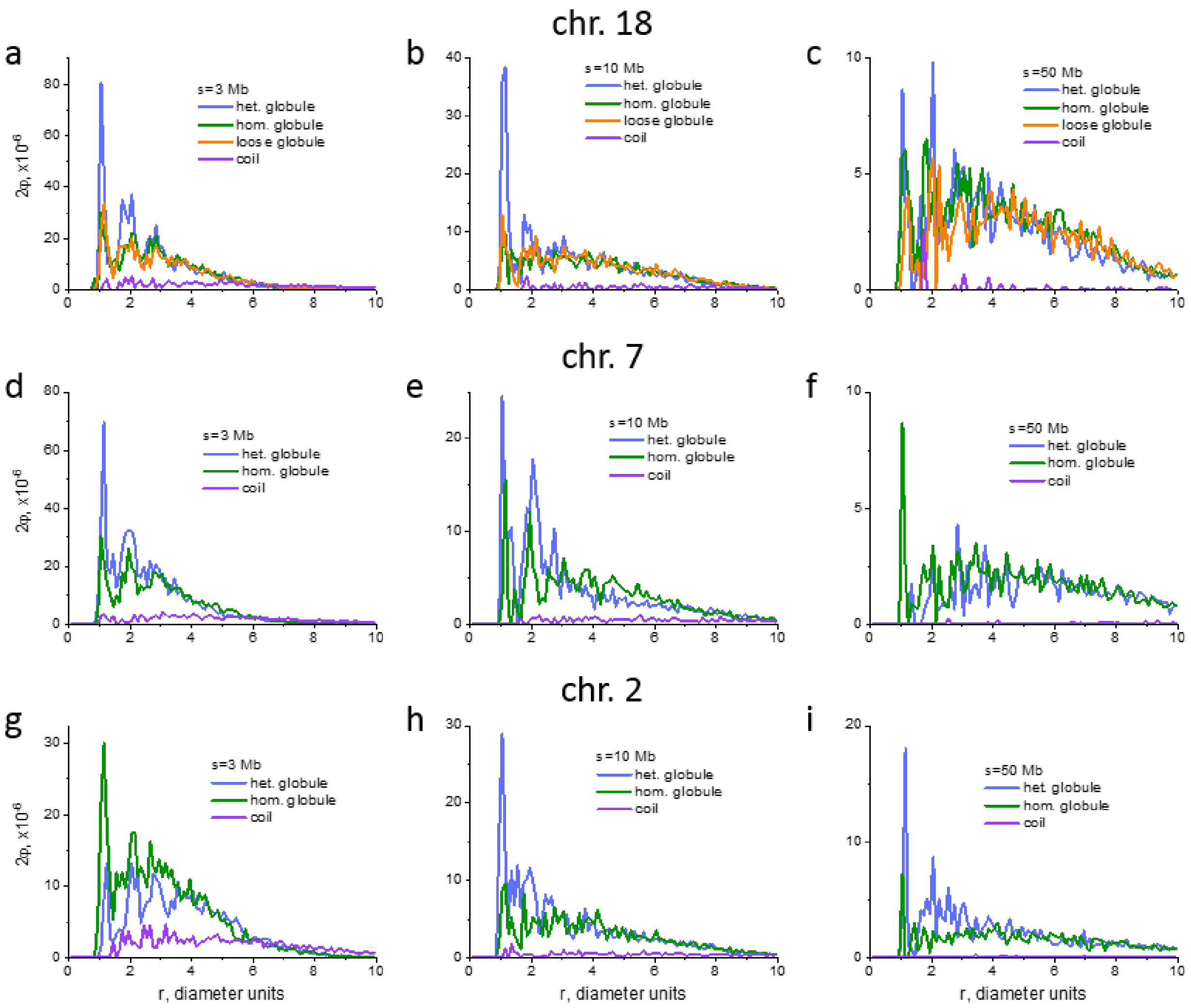

Figure S8. Quantification of IR-recurrent damage heterogeneity in different mouse chromosomes. Damage heterogeneity function $\varphi_{i,j}(r; D)$ for γ-radiation. D=5 Gy. $\varphi_{i,j}(r; D)$ was calculated for different models of chromosomes 18, 7, 2. Locus i is the position of the I-SceI recognition site, r is the distance between locus i and damaged locus j with DSB of any origin, spontaneous or IR-induced. $2\cdot\varphi_{i,j}(r; D)$ gives the density of damaged loci per cell. The results are given for individual j corresponding to genomic separations s from 3 to 50 Mb. (**a** – **c**) Chromosome 18, position of the I-SceI recognition site i=70.7 Mb. (**a**) s=3 Mb, j=67.7 Mb; (**b**) s=10 Mb, j=60.7 Mb; (**c**) s=50 Mb, j=20.7 Mb. (**d** – **f**) Chromosome 7, i=31.3 Mb. (**d**) s=3 Mb, j=28.3 Mb; (**e**) s=10 Mbp, j=21.3 Mb; (**f**) s=50 Mb, j=81.3 Mb. (**g** – **i**) Chromosome 2, i=180.2 Mb. (**g**) s=3 Mb, j=177.2 Mb; (**h**) s=10 Mb, j=170.2 Mb; (**i**) s=50 Mb, j=130.2 Mb. Structure models: heteropolymer globule (abbreviated as het. globule), homopolymer globule (hom. globule), loose globule (only for chromosome 18), self-avoiding polymer coil (coil).

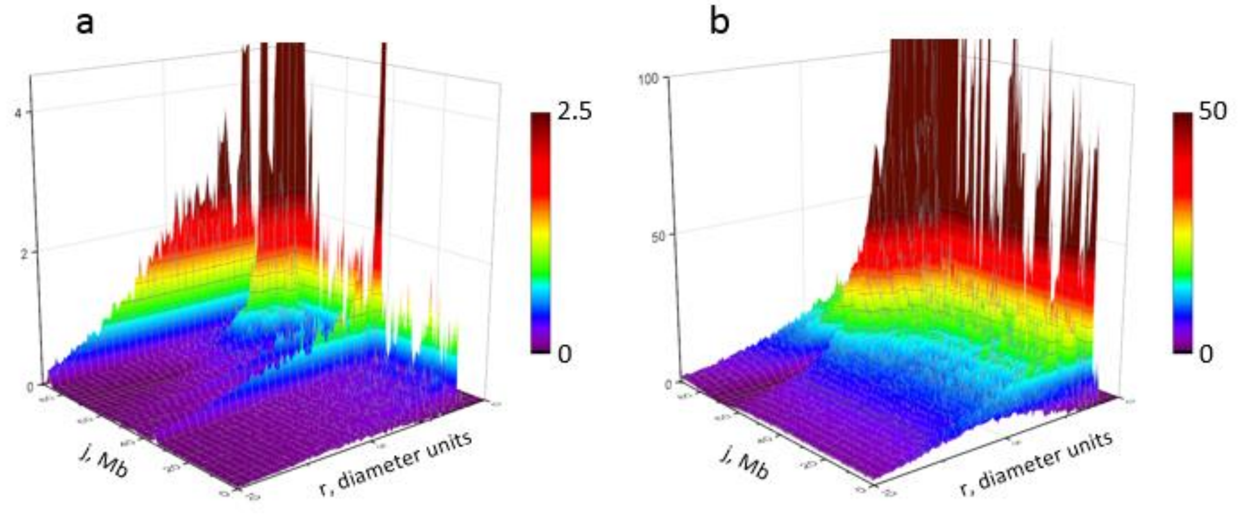

Figure S9. Damage proximity function $\varphi_{i,j}(r;D)$ for chromosome 18 after low and high doses of gamma-irradiation. (**a**) D=0.1 Gy; (**b**) D=15 Gy. The locus i is the position of the I-SceI recognition site, 70.7 Mb. r is the distance between the locus i and damaged locus j with DSB of any origin, spontaneous or IR-induced.

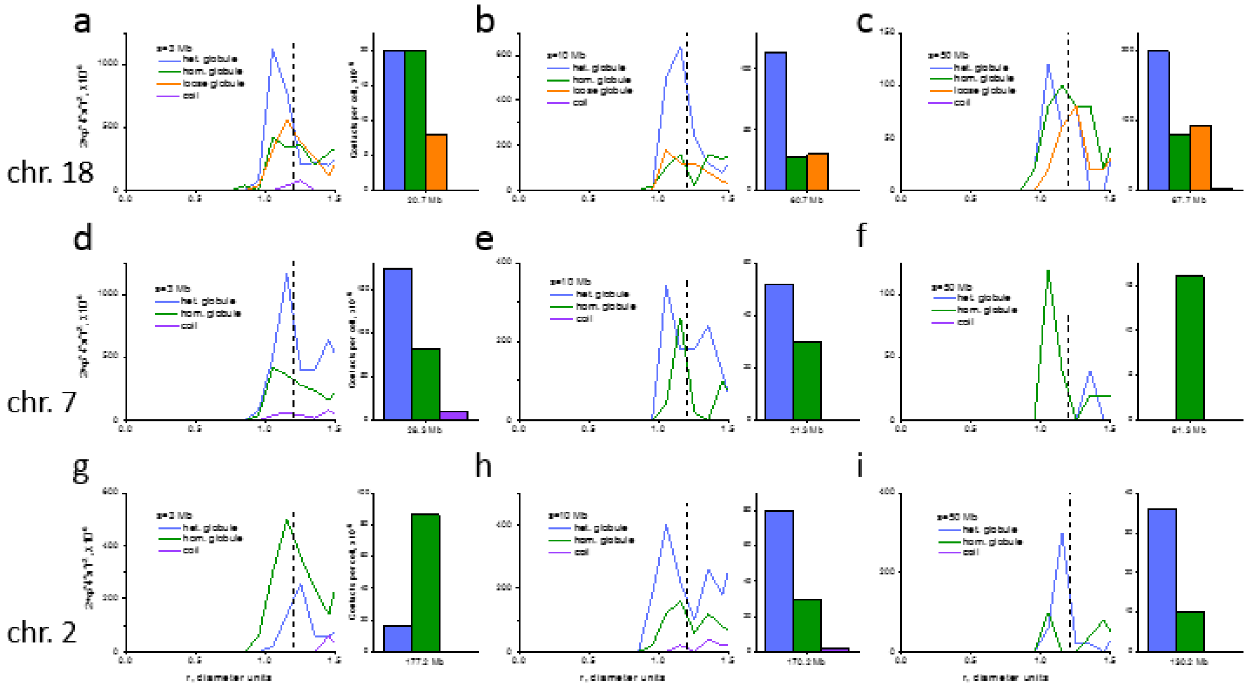

Figure S10. Lesion spatial heterogeneity distribution and IR-recurrent intrachromosomal lesion contacts. The function $\varphi_{i,j}(r;D)$, multiplied by $4\pi r^2 dr$ is the number of damaged loci i and j at distance r, r+dr from each other per chromosome. The integral of $\varphi_{i,j} 4\pi r^2 dr$ over the contact volume, from 0 to $R_{cont,CA}$=1.2 d, gives the number of contacting lesions i and j per chromosome. Multiplying by 2, one gets the number of contacting lesions per cell. Locus i contains a recurrent DSB and locus j contains DSBs formed by IR at dose D and spontaneous DSBs. The columns are the contact frequencies obtained from $\varphi_{i,j}(r;D)$ for different polymer models of chromosomes 2, 7, 18. Multiplying the number of contacts by the probability of contact-exchange, gives the number of translocation breakpoints between i and j loci per cell. The abbreviations for structure types are the same as in Figure S5. (**a** – **c**) Chromosome 18. (**d** – **f**) Chromosome 7. (**g** – **i**) Chromosome 2. (**a**, **d**, **g**) Genomic separation s=3 Mb. (**b**, **e**, **h**) s=10 Mb. (**c**, **f**, **i**) s=50 Mb.

At large distances, the distributions for hetero- and homopolymer globules and even for a loose globule (but not for a coil) reveal the features of universality for different chromosomes (Figure S7, Figure S8). For chromosome 18, this is the case for all genomic separations (s) and distances (r) greater than 2 – 3 subunit diameters; for chromosomes 2 and 7, all s and r > 3 – 4d. For relatively large genomic separations, 10 Mb and higher, function $\varphi_{i,j}(r;D)$ coincides for all globular models of all three chromosomes considered.

At short distances, the spatial distribution of damaged loci $\varphi_{i,j}(r; D)$ for various polymer models of each chromosome and for different chromosomes (Figure S8, Figure S10) demonstrates the impact of structural state on distribution of distances between lesions. Spatial heterogeneity of DNA damage in the form of distance distribution in the short range determines the frequency of lesion contacts and translocation breakpoints (Figure S10).

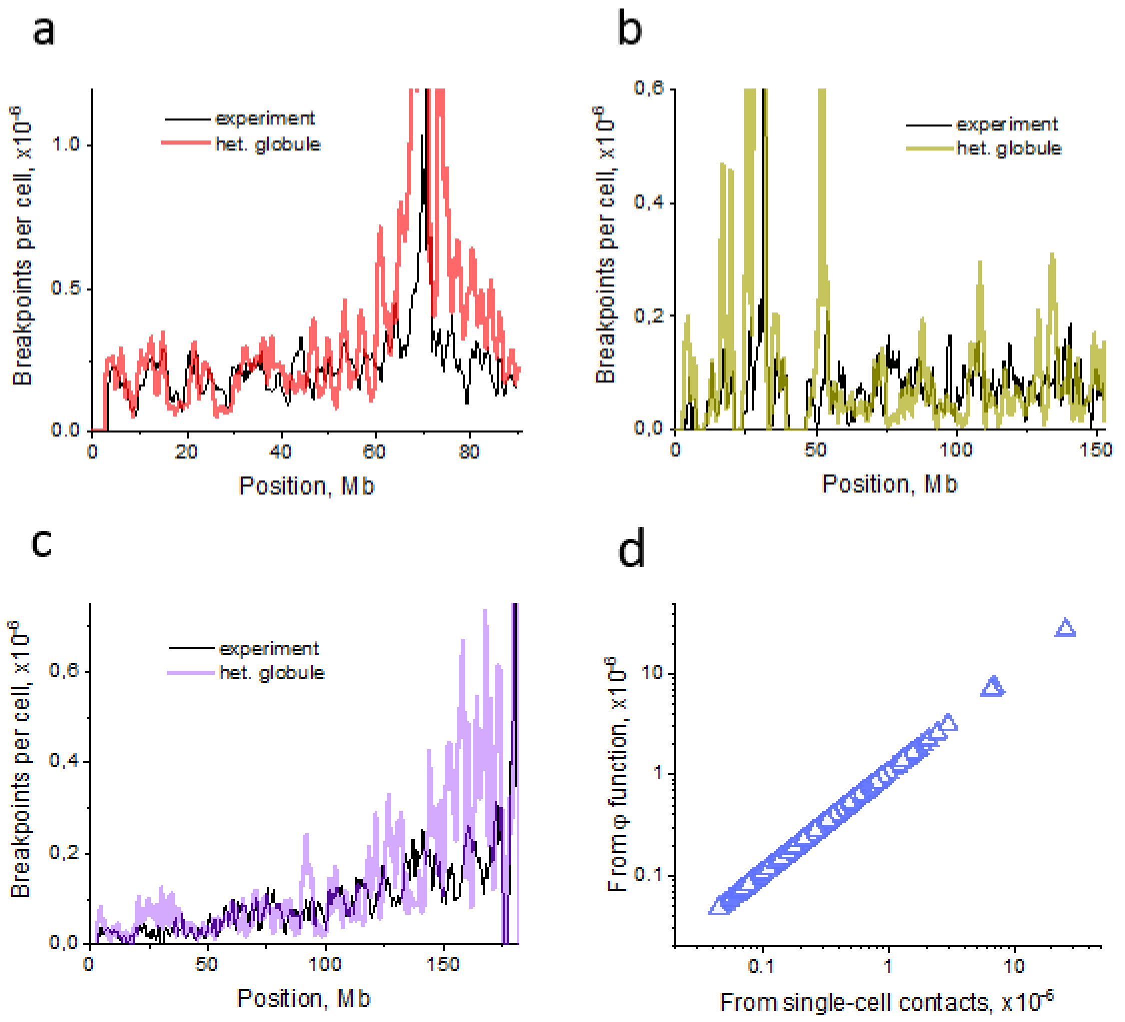

Figure S11. Prediction of breakpoint distribution with the incorporation of spatial heterogeneity information. Heteropolymer model of chromosomes, D=5 Gy. (**a**) Chromosome 18; (**b**) chromosome 7; (**c**) chromosome 2. (**d**) Correlation between breakpoints per cell predicted for chromosome 18 by two methods: from single-cell contacts and from damage spatial heterogeneity function $\varphi_{i,j}(r;D)$. When distances of Hi-C contact and damage colocalization for CA formation are equal Pearson's correlation equals unity.

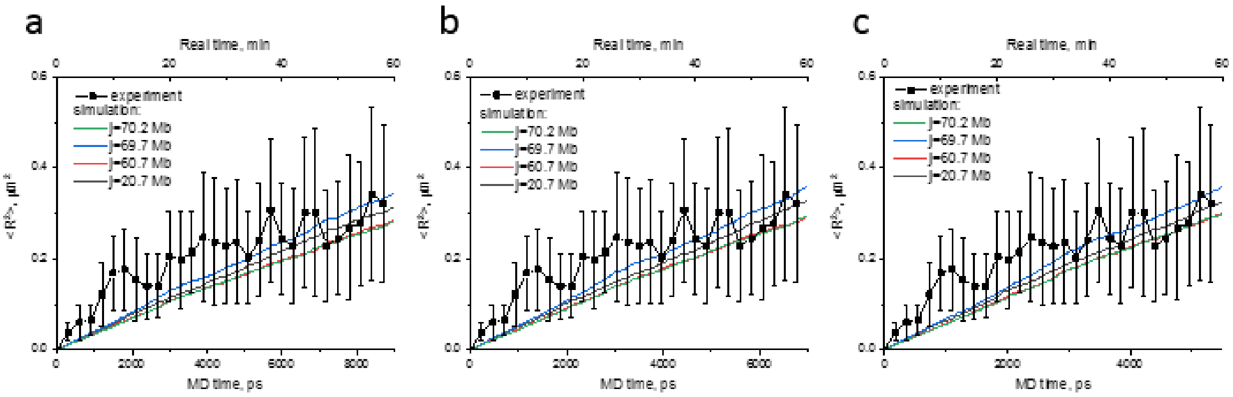

Figure S12. Conversion of the internal spatial and temporal scales of the simulations to the real scales. In all panels: upper X axis is the real time scale for experiment [42], lower X axis is the internal time scale in molecular dynamics simulations. The simulations are for chromosome 18 (heteropolymer globule). The simulated data on mean squared displacement are presented for 4 loci with various genomic separations from I-SceI site, 0.5 to 50 Mb. Locus positions j are shown in the legends. The panels correspond to different subunit diameters in real units. (**a**) d=175 nm; (**b**) d=200 nm; (**c**) d=225 nm.

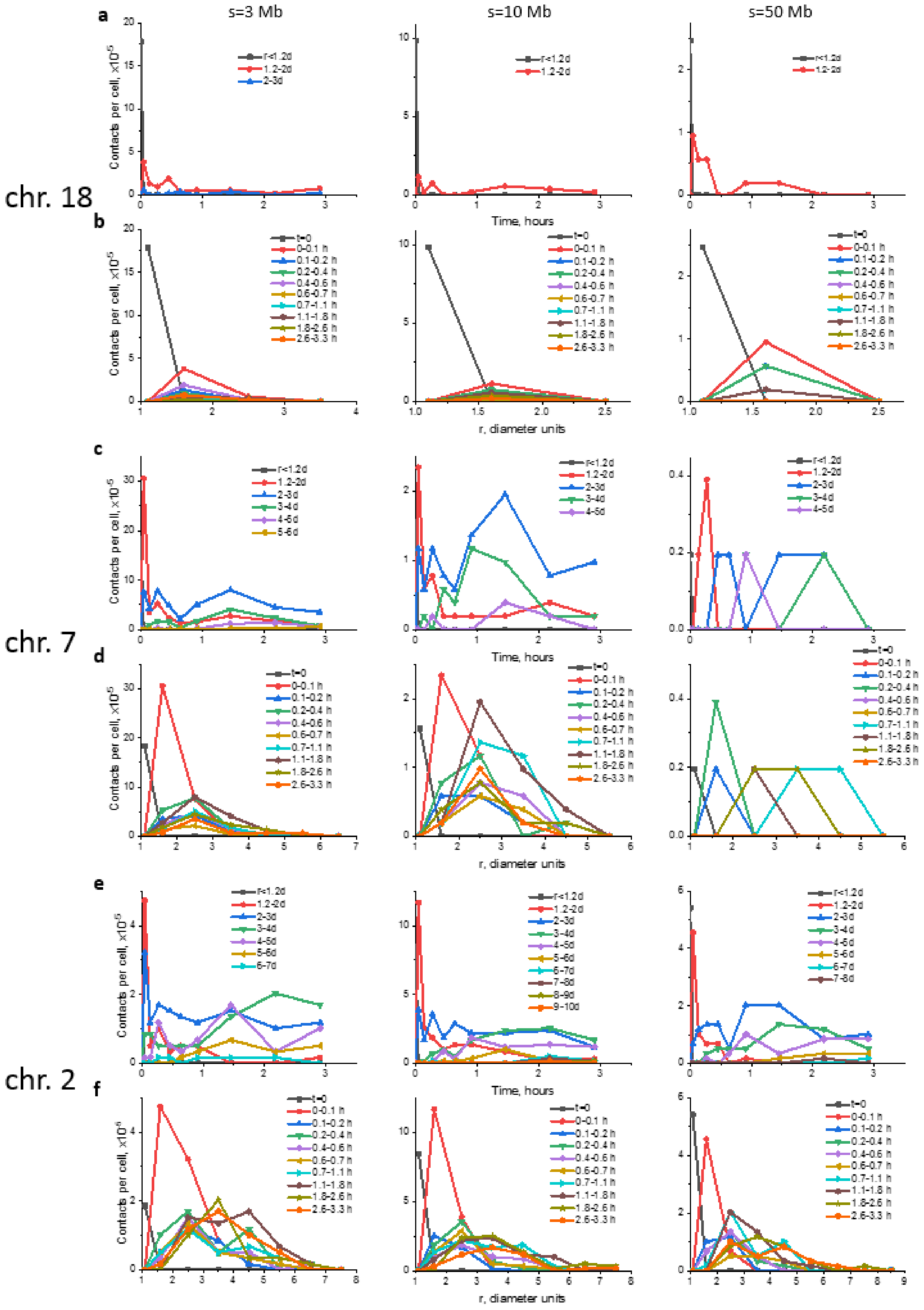

Figure S13. The dynamics of lesion contact formation is heterogeneous among chromosomes. The dose of γ-radiation D=5 Gy. For chromosomes 2, 7 and 18, the calculated dynamics of the formation of contacts with the I-SceI recognition site is shown for three IR-damaged loci of chromosomes located at a genomic separations s=3, 10 and 50 Mb. The figure shows a function

describing the number of contacts per cell formed in the time interval (t, t+Δt) from loci that at the initial time were at a distance of r, r+Δr. (**a**, **b**) Chromosome 18; (**c**, **d**) chromosome 7; (**e**, **f**) chromosome 2. (**a**, **c**, **e**) The number of contacts per cell formed in the time interval t, t+Δt at different initial distances between loci (in element diameter units). (**b**, **d**, **f**) The number of contacts of damaged loci as a function of the initial distance between loci formed at different time intervals.

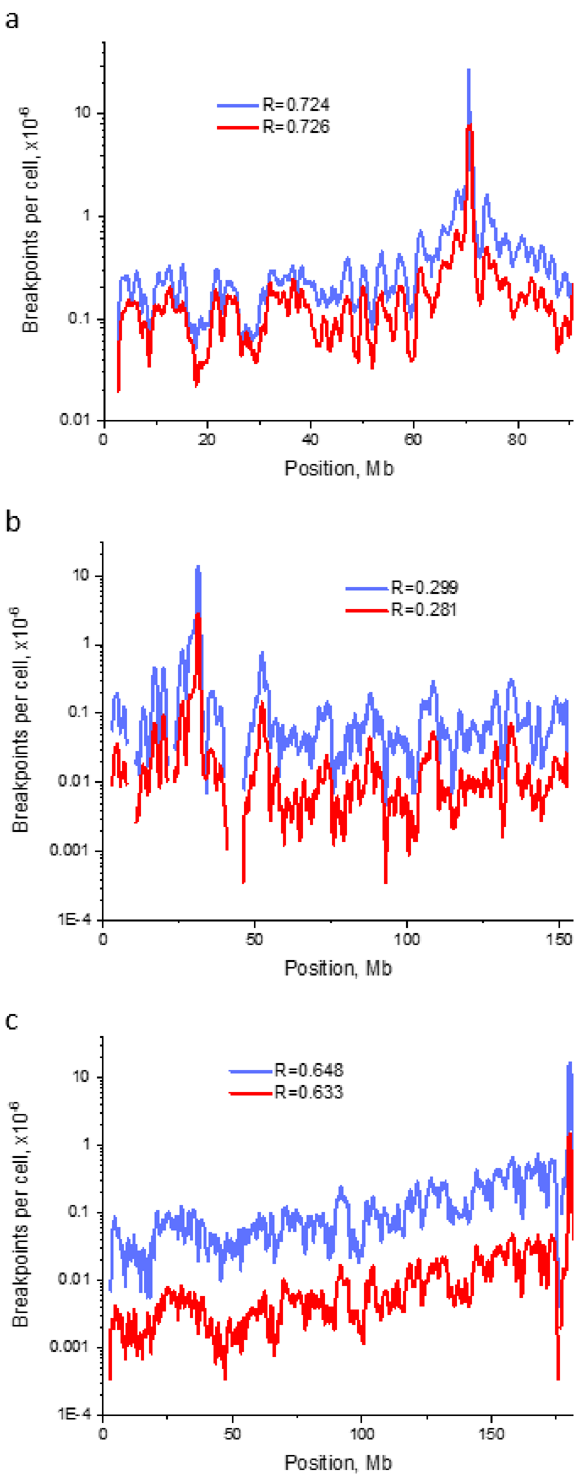

Figure S14. The shape for contact-first breakpoint distributions obtained by different mechanisms. Blue curves: experiment fitting by the contact-first mechanism (Figure 3). Red curves: aberrations formed at t=0, experiment fitting with the breakage-first mechanism (Figure 9). Pearson's correlations between the simulated distributions and experiment for chromosomes 18, 7, 2 [21] are shown in the panels.

Correlations between simulated blue and red distributions for each chromosome indicating the level of similarity of distribution shape: (**a**) chromosome 18. R=0.953 (without 1 Mb area around I-SceI site). (**b**) Chromosome 7. R=0.995 (without area 30.8-32.2 Mb around I-SceI site). (**c**) Chromosome 2. R=0.993 (without area 179.6-181.2 Mb around I-SceI site).

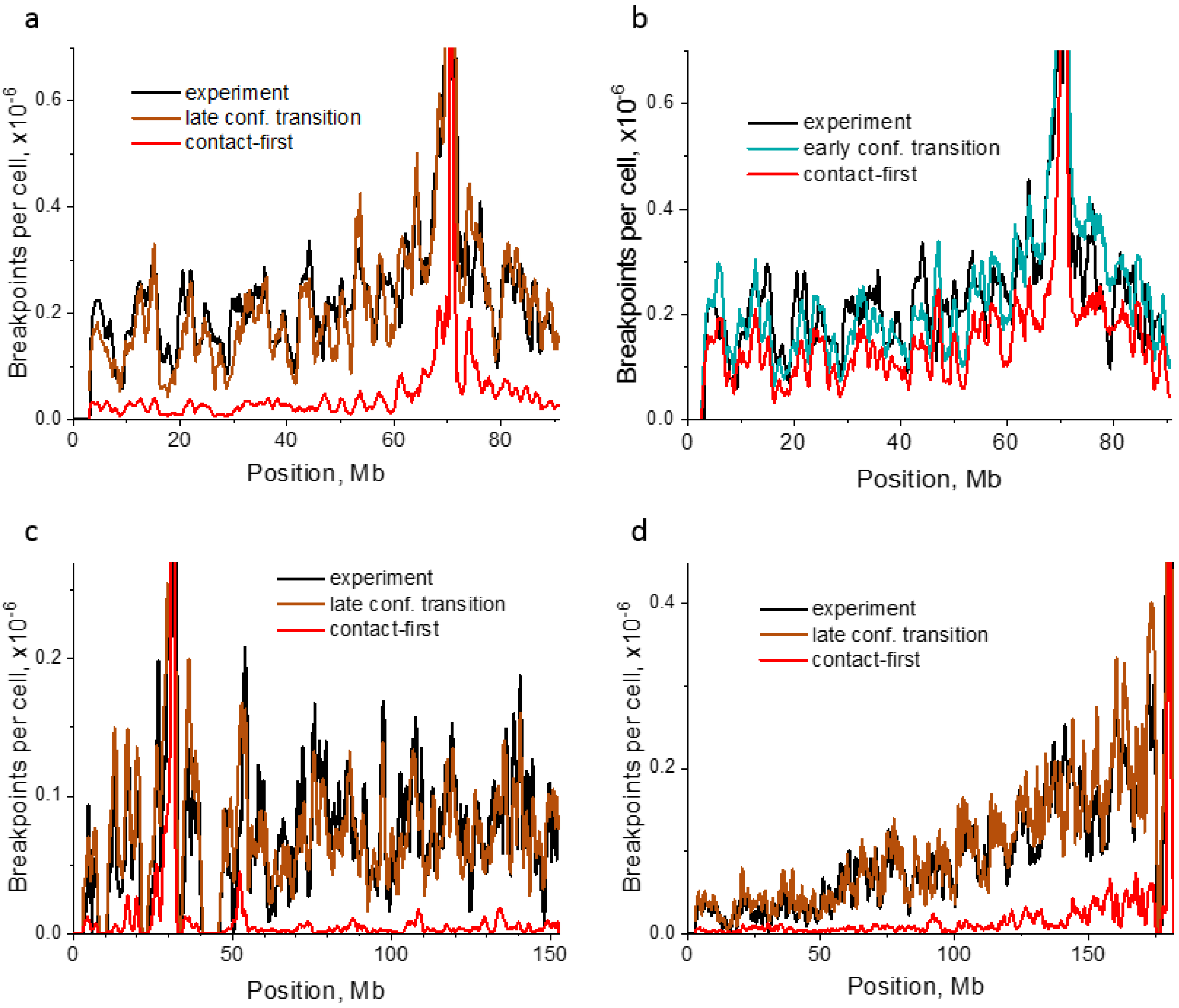

Figure S15. Distribution of breakpoints in mouse chromosomes of 2, 7, 18 with contribution of conformational transitions. (**a**, **b**) Chromosome 18. (**a**) Calculation for the scenario of late conformational transition in comparison with the experiment [21]. Chi-square=7.99, R=0.875. (**b**) The calculation for the scenario of early conformational transition. Chi-square=12.46, R=0.774. (**c**) chromosome 7, late conformational transition. Chi-square=7.92, R=0.705. (**d**) chromosome 2, late conformational transition. Chi-square=8.40, R=0.858. The red curves in all panels indicate the contribution of CAs formed by the contact-first mechanism to total breakpoint distributions.

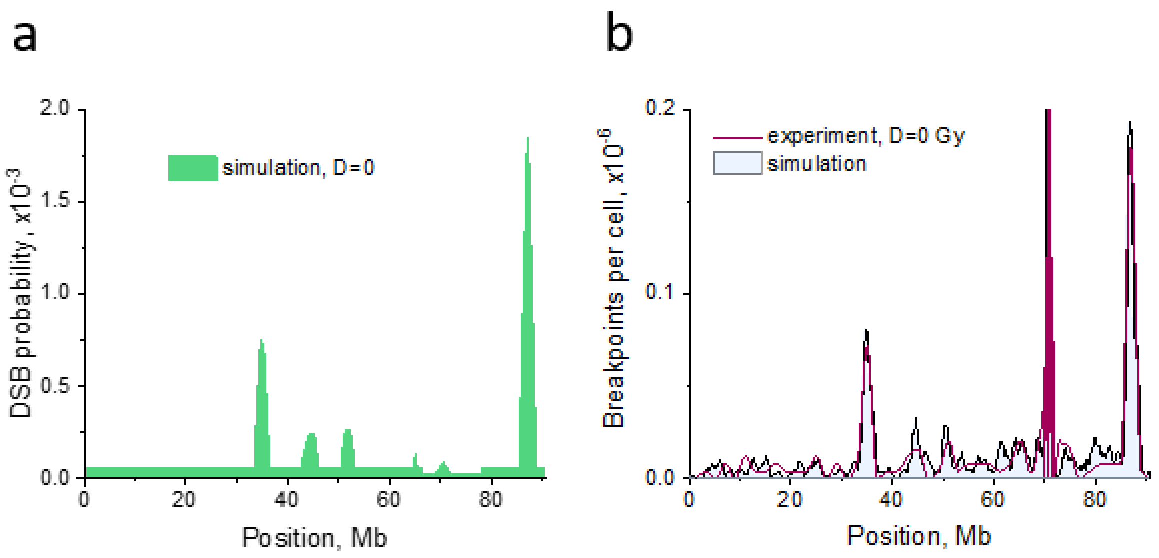

Figure S16. Simulation of spontaneous DSB distribution in chromosome 18. Heteropolymer globule model. (**a**) The reconstructed probability of a spontaneous DSB in the 100kbp subunit $\Gamma_{sp}(1,j)$. (**b**) Spontaneous breakpoint distribution compared to experiment [21].

Table S1. Comparison between compactness (number of contacts) between the chromosomes studied. Heteropolymer globule model.

Quantity (per chromosome, s>1)	Chromosome			Ratio for chromosome pair		
	18	7	2	7 : 18	2 : 18	2 : 7
Total contacts	3078	4431	4826	1.44	1.57	1.09
Subunit pairs, x10^6	0.41	1.16	1.65	2.83	4.02	1.42
**Contacts per subunit pair, x10^{-3}**	**7.51**	**3.82**	**2.92**	**0.51**	**0.39**	**0.76**
Contacts with I-SceI site	6.90	4.96	5.23	0.72	0.76	1.05
Number of subunits	905	1523	1815	1.68	2.01	1.19
**Contacts with I-SceI site per subunit, x10^{-3}**	**7.62**	**3.26**	**2.88**	**0.43**	**0.38**	**0.88**

Table S2. Comparison between compactness (number of contacts) between the chromosomes studied. Experiment [21].

Quantity (absolute counts, s>1)	Chromosome			Ratio for chromosome pair		
	18	7	2	7 : 18	2 : 18	2 : 7
Total contacts, x10^6	4.17	6.30	9.56	1.51	2.29	1.52
Subunit pairs, x10^6	0.41	1.16	1.65	2.83	4.02	1.42
**Contacts per subunit pair**	**10.17**	**5.43**	**5.79**	**0.53**	**0.57**	**1.07**
Contacts with I-SceI site, x10^3	9.57	8.39	10.17	0.88	1.06	1.21
Number of subunits	905	1523	1815	1.68	2.01	1.19
**Contacts with I-SceI site per subunit**	**10.57**	**5.51**	**5.60**	**0.52**	**0.53**	**1.02**

Tables S1 and S2 demonstrate different degrees of condensation of three chromosomes studied for both the heteropolymer globule model and the experiment [21]. Two criteria are used: the total number of contacts (which is related to compactness of a chromosome as a whole) and the number of contacts with I-SceI recognition site (relevant for CAs studied in the present work). Since these three chromosomes have different lengths, they would have different numbers of contacts even if they were equally compact. Thus, for interchromosomal comparison, it makes sense to use the normalized values: for the total number of contacts, divided by the number of subunit pairs; for the number of contacts with I-SceI site, divided by the chromosome length. The corresponding lines in Tables S1 and S2 are in bold. These two values are close to each other for each of the three chromosomes and differ between chromosomes markedly. Both criteria suggest that chromosome 18 is more condensed than chromosomes 2 and 7, and chromosomes 7 and 2 are condensed roughly equally. This conclusion is valid for the experiment as well as for the model.
